# JAK–STAT inhibition impairs K‐RAS‐driven lung adenocarcinoma progression

**DOI:** 10.1002/ijc.32624

**Published:** 2019-09-10

**Authors:** Julian Mohrherr, Marcel Haber, Kristina Breitenecker, Petra Aigner, Stefan Moritsch, Viktor Voronin, Robert Eferl, Richard Moriggl, Dagmar Stoiber, Balázs Győrffy, Luka Brcic, Viktória László, Balázs Döme, Judit Moldvay, Katalin Dezső, Martin Bilban, Helmut Popper, Herwig P. Moll, Emilio Casanova

**Affiliations:** ^1^ Department of Physiology, Center of Physiology and Pharmacology & Comprehensive Cancer Center (CCC) Medical University of Vienna Vienna Austria; ^2^ Ludwig Boltzmann Institute for Cancer Research (LBI‐CR) Vienna Austria; ^3^ Institute of Cancer Research Medical University of Vienna & Comprehensive Cancer Center (CCC) Vienna Austria; ^4^ Institute of Animal Breeding and Genetics University of Veterinary Medicine Vienna Austria; ^5^ Medical University of Vienna Vienna Austria; ^6^ MTA TK Lendület Cancer Biomarker Research Group, Institute of Enzymology, and Second Department of Pediatrics Semmelweis University Budapest Hungary; ^7^ Diagnostic & Research Institute of Pathology Medical University of Graz Graz Austria; ^8^ Division of Thoracic Surgery, Department of Surgery & Comprehensive Cancer Center (CCC) Medical University of Vienna Vienna Austria; ^9^ Department of Biomedical Imaging and Image‐guided Therapy, Division of Molecular and Gender Imaging Medical University of Vienna Vienna Austria; ^10^ Department of Tumor Biology, National Korányi Institute of Pulmonology Semmelweis University Budapest Hungary; ^11^ Department of Thoracic Surgery National Institute of Oncology and Semmelweis University Budapest Hungary; ^12^ SE‐NAP Brain Metastasis Research Group, 2nd Department of Pathology Semmelweis University Budapest Hungary; ^13^ First Department of Pathology and Experimental Cancer Research Semmelweis University Budapest Hungary; ^14^ Department of Laboratory Medicine Medical University of Vienna Vienna Austria; ^15^ Core Facilities Medical University of Vienna Vienna Austria

**Keywords:** non‐small cell lung cancer, lung adenocarcinoma (AC), Kirsten rat sarcoma viral proto‐oncogene (K‐RAS), Janus kinase (JAK), ruxolitinib, cell‐line derived xenografts, genetically engineered mouse models, tumor microenvironment (TME), tumor promoting inflammation

## Abstract

Oncogenic K‐RAS has been difficult to target and currently there is no K‐RAS‐based targeted therapy available for patients suffering from K‐RAS‐driven lung adenocarcinoma (AC). Alternatively, targeting K‐RAS‐downstream effectors, K‐RAS‐cooperating signaling pathways or cancer hallmarks, such as tumor‐promoting inflammation, has been shown to be a promising therapeutic strategy. Since the JAK–STAT pathway is considered to be a central player in inflammation‐mediated tumorigenesis, we investigated here the implication of JAK–STAT signaling and the therapeutic potential of JAK1/2 inhibition in K‐RAS‐driven lung AC. Our data showed that JAK1 and JAK2 are activated in human lung AC and that increased activation of JAK–STAT signaling correlated with disease progression and K‐RAS activity in human lung AC. Accordingly, administration of the JAK1/2 selective tyrosine kinase inhibitor ruxolitinib reduced proliferation of tumor cells and effectively reduced tumor progression in immunodeficient and immunocompetent mouse models of K‐RAS‐driven lung AC. Notably, JAK1/2 inhibition led to the establishment of an antitumorigenic tumor microenvironment, characterized by decreased levels of tumor‐promoting chemokines and cytokines and reduced numbers of infiltrating myeloid derived suppressor cells, thereby impairing tumor growth. Taken together, we identified JAK1/2 inhibition as promising therapy for K‐RAS‐driven lung AC.

## Abstract 2

**What's new?**

A drug that inhibits the JAK–STAT pathway may score a hit against K‐RAS driven lung cancer. Here, the authors Investigated the JAK STAT pathway as a possible target in lung adenocarcinoma because of its role in inflammation‐mediated tumorigenesis. First, they showed that JAK1 and JAK2 are both activated in lung adenocarcinoma patients with oncogenic mutations in K‐RAS. Next, they treated the tumors with ruxolitinib, which inhibits JAK1/2. The drug successfully slowed tumor proliferation and progression in immunocompetent mouse models. Furthermore, treatment with ruxolitinib reduced the tumor‐promoting factors present in the microenvironment.

**Abbreviations**
AC: adenocarcinoma
ACTB: actin beta
ANOVA: one‐way analysis of variance
CXCL1: chemokine (C‐X‐C motif) ligand 1
EGFR: epidermal growth factor receptor
GO: gene ontology
GSEA: gene set enrichment analysis
IL: interleukin
JAK: Janus kinase
K‐RAS: Kirsten rat sarcoma viral proto‐oncogene
MDSCs: myeloid derived suppressor cells
mRNA: messenger RNA
NKs: natural killer cells
PD‐L1: programmed cell death 1 ligand 1
RNA‐seq: RNA sequencing
STAT: signal transducer and activator of transcription
TBP: TATA‐box binding protein
TME: tumor microenvironment
TNF: tumor necrosis factor
VEGF: vascular endothelial growth factor
WT: wild‐type

## Introduction

Lung cancer is still the leading cause of cancer‐related deaths worldwide in both men and women.^1^ Histologically, lung cancer can be subdivided into small cell lung cancer and non‐small cell lung cancer, with lung adenocarcinoma (AC) being the most abundant among the non‐small cell lung cancer subtype.^2^ Roughly 50% of lung ACs harbor oncogenic mutations in the V‐Ki‐ras2 Kirsten rat sarcoma viral oncogene homolog (K‐RAS) or epidermal growth factor receptor (EGFR).^3^, ^4^ While EGFR tyrosine kinase inhibitors, such as erlotinib, gefitinib or afatinib, are part of the clinical routine to treat *EGFR*‐mutated lung AC patients,^5^ the development of K‐RAS inhibitors has been less successful so far, with no K‐RAS‐based targeted therapy being clinically approved.^6^ Hence, targeting alternative tumor drivers fueling K‐RAS‐mediated lung tumorigenesis such as tumor‐promoting inflammation and K‐RAS downstream effector pathways are considered to be a promising treatment strategy.^6^, ^7^

Given the pivotal role of Janus kinase (JAK) activation in tumor‐promoting inflammation and its oncogenic functions, JAKs represent attractive therapeutic targets in tumorigenesis.^8^ The JAK family is composed of four members (JAK1, JAK2, JAK3 and tyrosine kinase 2) and couples extracellular stimuli with gene transcription. Upon cytokine binding to their cognate receptors, JAKs become activated and phosphorylate downstream signal transducer and activator of transcription (STAT) molecules, ultimately leading to nuclear translocation and transcription of various target genes involved in cell cycle regulation, angiogenesis, apoptosis, tumor invasion and metastasis.^9^ JAK signaling either promotes or suppresses tumorigenesis in a tumor cell intrinsic manner. Indeed, *K‐RAS*‐mutated lung AC cells secrete pro‐inflammatory cytokines including interleukin (IL)‐6 that activate JAK1 and JAK2 *via* glycoprotein 130 in an autocrine loop, thereby promoting tumor cell survival.^7^, ^10^, ^11^ On the other hand, genetic deletion of STAT3, a key signaling mediator of JAK1/2, enhances K‐RAS‐driven lung tumorigenesis, and activation of the interferon/JAK/STAT axis induces cell apoptosis and suppresses tumorigenesis in various experimental tumor models.^12^, ^13^, ^14^, ^15^, ^16^

In addition to these tumor‐cell intrinsic effects, JAK signaling is also substantially involved in shaping the tumor microenvironment (TME) by modulating T, natural killer (NK) and dendritic cell functions.^17^ As in tumor cells, JAK activation in the TME and its role in tumorigenesis is controversially discussed. While JAK1/2 inhibition impairs NK‐cell function and NK mediated antitumor immunity and hence indirectly facilitates tumor metastatic spread,^18^ targeting the JAK–STAT axis has also been proposed as a potential effective strategy to reduce levels of tumor‐promoting immunosuppressive myeloid derived suppressor cells (MDSCs), thereby counteracting malignant progression.^19^ Furthermore, IL6/JAK/STAT3, as well as interferon‐γ/JAK/STAT1 signaling pathways have also been described to induce programmed cell death 1 and or programmed cell death 1 ligand 1 (PD‐L1) expression and, therefore, to promote tumor immune escape.^20^, ^21^ Taken together, these reports indicate that JAKs may have opposite functions in tumorigenesis depending on the cellular context.

Clinical trials investigating JAK inhibition in particular for *EGFR*‐mutated lung cancers are ongoing. These studies combine the JAK inhibitors ruxolitinib, itacitinib and AZD4205 with the EGFR tyrosine kinase inhibitors erlotinib (NCT02155465), afatinib (NCT02145637) and osimertinib (NCT02917993, NCT03450330). By contrast, JAK targeting in *K‐RAS*‐mutated lung ACs has not been thoroughly investigated so far. Notably, a recent clinical trial showed positive results when combining ruxolitinib and chemotherapy in non‐small cell lung cancer patients, but the K‐RAS mutation status is not available (NCT02119650). Thus, to complement these clinical studies, we addressed the role of JAK1/2 in K‐RAS‐driven lung AC pathogenesis. By using human tumor samples and experimental mouse models, we show that JAK1/2 are activated in K‐RAS‐driven lung AC and promote tumor growth and progression by establishing a protumorigenic TME. Importantly, pharmacological inhibition of JAK1/2 with the tyrosine kinase inhibitor ruxolitinib reverts the protumorigenic TME and impairs tumorigenesis.

## Materials and Methods

### Human data

For gene expression analysis, we took advantage of the publicly available Illumina microarray data set, which contains biopsies of *K‐RAS*‐mutated lung AC tissue and adjacent healthy tissue (GSE75037).^22^ To determine *JAK1* and *JAK2* mRNA expression levels, we used probes as depicted in Table S1. As a second cohort, we used gene expression data from *K‐RAS‐*mutated lung AC patients included in the Cancer Genome Atlas (*n* = 154).^23^

Gene set enrichment analysis (GSEA) was performed according to the provider's protocol and permutations were set to 1,000.^24^ Gene sets were downloaded from the Molecular Signature Database (http://software.broadinstitute.org/gsea/msigdb/index.jsp). To determine the prognostic value of chemo/cytokine related genes upon ruxolitinib treatment for human lung ACs pathogenesis, we used the KM‐Plotter online tool.^25^ In addition, gene expression data of *K‐RAS*‐mutated lung AC patients with available survival data included in the Cancer Genome Atlas were analyzed ( *n* = 150).^23^ We have run the analysis by evaluating all possible cutoff values and the best performing value was used as the cutoff in the final analysis. False discovery rate was computed to correct for multiple testing, and only results having a false discovery rate below 1% was accepted as statistically significant. Analysis was performed using the mean expression of the genes included in the multigene signature, and all utilized Probe‐IDs are shown in Table S2.

For immunohistochemical analysis, we generated tissue microarrays from two independent lung AC patient cohorts. Cohort A includes biopsies from lung AC patients where information about the mutational *K‐RAS* status was not available (pJAK1 *n* = 303, pJAK2 *n* = 318). Cohort B consists of biopsies of lung AC patients positive for *K‐RAS* mutation (pJAK1 *n* = 26, pJAK2 *n* = 24). Analysis was done with prior approval by the institutional ethical committee. For detection of JAK1 or JAK2 tyrosine phosphorylation the tissue microarrays were stained with a pJAK1 (1:200, Y1022, #PA537617, Thermo Fisher Scientific, Waltham, MA) and a pJAK2 (1:200, Y1007 and Y1008, #32101, Abcam, Cambridge, UK) antibody. Histoscores were determined according to staining intensities and percentage of positive tumor cells by a board‐certified pathologist (H.P.).^26^

### Cell lines

The human *K‐RAS*‐mutated lung AC cell lines A549 and A427 were grown in RPMI medium (Gibco, Grand Island, NY) supplemented with 10% FBS, 2 mM glutamine (Gibco), penicillin (50 U/ml, Gibco) and streptomycin (50 μg/ml, Gibco). A549 and A427 cell lines were obtained from American Type Culture Collection, and cells were profiled for authentication (Eurofins Genomics, Luxembourg). Cells were tested to be free of mycoplasma contaminations twice a year. For experiments, cells were harvested at ~70% confluency, and the number of viable cells was determined using a CASY counter (Roche, Basel, Switzerland).

### Animal husbandry and experiments

For subcutaneous xenograft experiments using the *K‐RAS*‐mutated human lung AC cell lines A549 and A427, 1 × 10^6^ cells were mixed 1:2 with Matrigel (Corning, Corning, NY) and injected into the right and left flank of immunodeficient NOD scid gamma mice older than 6 weeks of age. Tumor volume was measured using a caliper and tumor volume was calculated using the formula [(length × width^2^) × 0.52] as previously established in our laboratory.^27^

*K*‐*ras* ^*LSL‐G12D*^ (K) mice as well as *K‐ras* ^*LSL‐G12D*^:*p53* ^*fl/fl*^ (KP) mice, both bred on a C57Bl/6 background, served as genetically engineered mouse models for autochthonous lung AC.^28^ Genotyping of the transgenes was performed using primers depicted in Table S3. Induction of lung tumors in these genetically engineered mouse models was achieved through intranasal inhalation of a Cre‐recombinase expressing adenovirus (Ad.Cre) in 8–12 week old mice using 2.5 × 10^7^ plaque‐forming units. Inhalation of K and KP mice with Ad.Cre triggers *K‐ras* ^*G12D*^ activation in lung epithelial cells resulting in formation of p53 expressing (K) or deficient (KP) lung ACs. Ad.Cre was purchased from the Viral Vector Core (University of Iowa). For treatment of mice with the JAK1/2 inhibitor ruxolitinib (LC Labs, Woburn, MA), the inhibitor was suspended in 0.5% (w/v) methylcellulose solution. Mice were treated *via* oral gavage with vehicle (ctrl, 0.5% Methylcellulose) or ruxolitinib (Ruxo) at 90 mg/kg twice a day (BID), 7 days per week for the indicated experimental time period. Treatment of mice was paused if any signs of distress occurred.^18^ All experimental protocols as well as animal maintenance and breeding described above, followed ethical guidelines and were approved by the Austrian Federal Ministry of Science, Research and Economy.

### Histology

At the experimental endpoint, subcutaneously engrafted tumor tissue or lung tissue was retrieved and fixed in 2% buffered formaldehyde at 4°C for 24 hr and embedded in paraffin after dehydration. Five micron sections were used for analysis. To determine tumor area, lung sections were stained with hematoxylin and eosin and subsequently scanned using TissueFaxs (TissueGnostics, Vienna, Austria) software. Tumor area and number were quantified using HistoQuest (TissueGnostics) software. Tumor grades were determined by a board‐certified pathologist Katalin Dezso (K.D.) according to DuPage *et al*.^28^ For immunohistochemistry, standard protocols were applied, using antibodies against pSTAT3 (1:200,Y705, #9145, Cell Signaling, Danvers, MA), KI67 (1:400, Cell Signaling #12202 or 1:200, #14‐5698‐82, eBioscience, San Diego, CA), cleaved caspase‐3 (1:200, Cell Signaling #9661), cluster of differentiation 31 (1:400, Cell Signaling #77699), PD‐L1 (1:200, Cell signaling #64988). For analysis of stained immunohistochemistry slides HistoQuest (TissueGnostics) software was used.

### RNA‐sequencing (RNA‐seq) analysis of total lungs and Gene Expression Omnibus data set analysis

For RNA‐seq analysis, K mice were treated 8 weeks post inhalation with Ad.Cre for four consecutive days with vehicle (0.5% Methylcellulose) or ruxolitinib (90 mg/kg BID). Additionally tumor harboring lungs from KP mice and lungs from wild‐type (WT) mice were included in the analysis. At the experimental endpoint, lungs were harvested and RNA was isolated using the RNeasy Kit (Qiagen, Venlo, Netherlands) following on column DNA digestion using RNase‐Free DNase (Qiagen). Library preparation and sequencing was carried out by the Core facility Genomics, Medical University of Vienna, Vienna, Austria. Briefly, sequencing libraries were prepared using the NEBNext® Ultra™ II RNA Library Prep Kit (NEB, Ipswich, MA) according to manufacturer's instructions and sequenced on a Illumina NextSeq500 platform in a 75 bp single‐read mode. RNA‐Seq data were mapped to the Mus musculus/mmc10 assembly of the murine genome using the STAR Aligner.^29^ Differential gene expression was analyzed using DESeq2.^30^

For GSEA, gene sets were downloaded from the Molecular Signature Database (http://software.broadinstitute.org/gsea/msigdb/index.jsp). For gene ontology (GO)‐molecular function analysis, the Enrichr web tool was used (http://amp.pharm.mssm.edu/Enrichr/enrich). The top 350 downregulated genes (based on log2 fold changes) in the ruxolitinib treated group were subjected to pathway analysis with Enrichr.^31^ To investigate the implications of ruxolitinib downregulated chemo/cytokine related genes for K‐RAS‐driven lung tumorigenesis, we retrieved all genes within the chemo/cytokine related GO gene sets GO0042379, GO0008009, GO0045236, GO0048020 and GO0005125. Data were analyzed using the web‐based online tool InteractiVenn,^32^ and for hierarchical clustering and heatmap illustration, we used the heatmapper.ca web tool.^33^ In order to check for the activation status of the JAK–STAT pathway and the expression of chemo/cytokine related genes in primary *K‐ras* ^*G12D*^‐mutated lung alveolar type II (K) cells, we analyzed the publically available GSE113146 data set.^27^

### RNA extraction and real‐time quantitative polymerase chain reaction

RNA isolation from the lungs was performed using TRIzol™ Reagent (Invitrogen, Carlsbad, CA) according to the manufacturer's protocol. Complementary DNA was transcribed using the RevertAid H Minus Reverse Transcriptase (Fermentas, Waltham, MA) kit. For detection of the transcript, the iTaq Universal SYBR Green reagent (Biorad, Hercules, CA) and a Biorad CFX Connect Real Time polymerase chain reaction system were utilized, using specific primers depicted in Table S4. For determination of relative gene expression ratios, the Pfaffl method was applied. Calculation of bestkeeper values was performed using actin beta (*ACTB*), *28S* and TATA‐box binding protein (*TBP*) as housekeeping genes.^34^

### Flow cytometric analysis of total lungs

At the experimental endpoint, *K‐RAS*‐mutated tumor harboring lungs were harvested, and the lung tissue was mechanically and enzymatically disrupted for cell isolation. Briefly, lungs were put into isolation medium (RPMI with 5% FBS) and minced with scissors. Minced pieces were then transferred into a falcon tube containing isolation medium supplemented with DNase I (50 U/ml, Sigma, St.Louis, MO) and Collagenase I (Gibco, 150 U/ml), and incubated for 1 hr at 37°C. Subsequently, the cell suspension was filtered through 70 and 40 μm cell strainers. For subsequent flow cytometric analysis, cells were stained with the antibodies indicated in Table S5 using a standard protocol. For identification of Th17 and regulatory T cells, the mouse Th17/Treg phenotyping kit (#560767, BD Pharmingen, Franklin Lakes, NJ) was used. All cells are CD45^+^. Stained samples were measured using the BD FACSCanto™ II system. For data analysis, the FlowJo software (TreeStar, Inc., Ashland, OR) was used.

### Statistical analysis

All values are presented as mean ± standard error of mean. For single outlier detection the Grubbs‐test was used, multiple outliers were defined when values were lower than Q1–1.5 × interquartile range or greater than Q3 + 1.5 × interquartile range (difference between the third quartile [Q3] and first quartile [Q1]). Subsequent statistical analyses were either performed utilizing Student's *t*‐test or Mann–Whitney *U*‐test calculated by GraphPad Prism 5.0 software. For multiple group comparison, one‐way analysis of variance (ANOVA) in combination with Tukey's multiple comparison test was used. For GO‐molecular function analysis, the results were ranked according to the adjusted *p*‐value. For Kaplan–Meier analysis, the Log‐rank test was used and false discovery rate was computed to combat for multiple hypothesis testing. For all graphs: *p < 0.05; **p < 0.01; ***p < 0.001.

## Results

### JAK signaling is activated in human *K‐RAS‐*mutated lung AC during tumor progression

**Figure 1:**
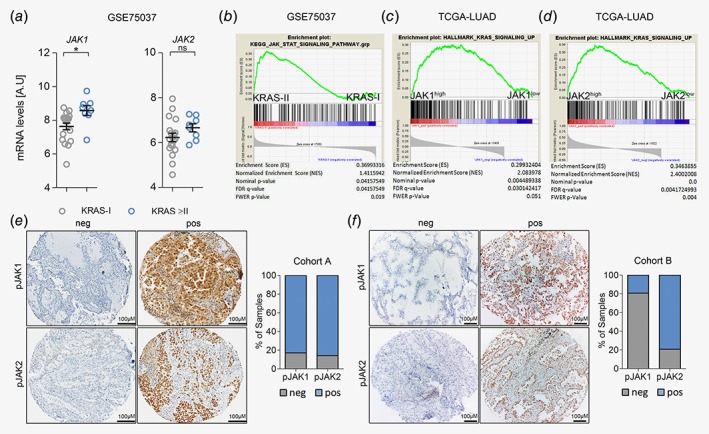
JAK‐mediated signaling is activated in progressing K‐RAS‐mutated human lung AC. (*a*) Graph showing relative *JAK1* and *JAK2* mRNA expression levels in human *K‐RAS*‐mutated lung AC tissue of stage I (*n* = 19) *versus* stage II or higher (≥II, *n* = 8). Data represent mean ± SEM, A.U (arbitrary units), Student's *t*‐test, *p < 0.05. (*b*) GSEA for Kyoto encyclopedia of genes and genomes‐JAK–STAT pathway signature geneset comparing human *K‐RAS*‐mutated tumors of stage I *versus* stage II, using tumor *versus* healthy parenchyma mRNA expression ratios. Data in (*a*) and (*b*) were retrieved from the Gene Expression Omnibus (GSE75037). (*c*) GSEA using gene expression data of the Cancer Genome Atlas‐LUAD cohort and HALLMARK_KRAS_SIGNALING_UP geneset, stratifying patients according to *JAK1* and (*d*) *JAK2* expression levels (*n* = 154). (*e*) Representative pictures of lung AC biopsies of patients included in cohort A (unknown *K‐RAS* mutation status; pJAK1 *n* = 303, pJAK2 *n* = 318) and (*f*) cohort B (*K‐RAS* mutated; pJAK1 *n* = 26, pJAK2 *n* = 24) with negative and positive staining reactions for pJAK1 and pJAK2 in tumor cells. Graphs represent percentage of positive and negative cases within the respective cohort. Staining intensities and percentage of positive tumor cells were determined by a board‐certified pathologist (H.P.). [Color figure can be viewed at http://wileyonlinelibrary.com]

To investigate the implication of *JAK1* and *JAK2* for the pathogenesis of human *K‐RAS*‐mutated lung AC, we took advantage of the publicly available mRNA expression data set GSE75037.^22^ Comparison of *JAK1* and *JAK2* mRNA expression levels of stage I classified *K‐RAS*‐mutated lung AC patients *versus* those with more advanced disease revealed increased *JAK1/2* expression levels in the latter group, which reached significance for *JAK1* (Fig. 1 *a*). In line with these data, we found activation of the JAK–STAT pathway in patients with more advanced lung AC (i.e., in stage II disease), as determined by GSEA (Fig. 1 *b*).^24^ Next, we confirmed these data in *K‐RAS* mutated lung AC patients from a different cohort published by the Cancer Genome Atlas.^23^ Indeed, we found a significant correlation of *JAK1* and *JAK2* mRNA expression levels with enrichment of K‐RAS signaling in lung AC patients (Figs. 1 *c* and 1 *d*). Moreover, we noticed a significant increase of *JAK1* and *JAK2* mRNA expression in tumors of late stage patients with oncogenic K‐RAS mutations compared to patients harboring K‐RAS WT tumors, which was not observed in early stage patients (Figs. S1 *a* and S1 *b*).

However, using mRNA expression data of bulk tumor tissue does not allow distinguishing between tumor and stromal cells. Hence, we performed immunohistochemistry for activated JAK1 and JAK2 in biopsies of lung AC patients from two independent cohorts (cohorts A&B, consisting of unselected and *K‐RAS* mutated samples, respectively). Indeed, JAK1 and JAK2 were activated/phosphorylated in tumor cells of lung AC patients as determined by a pathologist (H.P.). In the stroma, a positive reaction for JAK1 and JAK2 was mainly found in lymphocytes (Figs. 1 *e* and 1 *f*). Taken together, these data indicated that JAK signaling is active in human K‐RAS‐driven lung AC.

### JAK inhibition impairs human *K‐RAS*‐mutated lung AC cell growth *in vivo*

**Figure 2:**
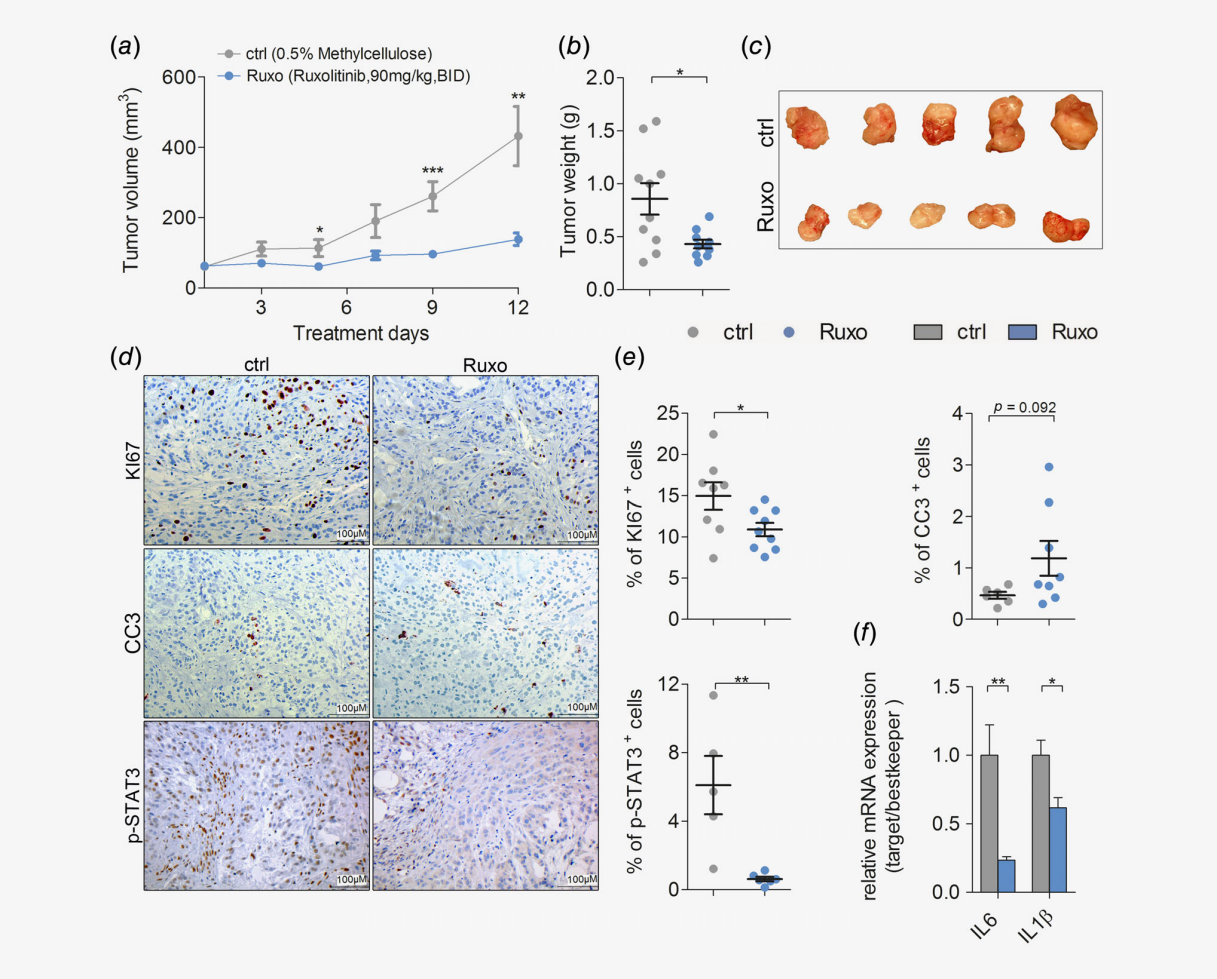
JAK inhibition impairs growth of human K‐RAS‐mutated lung AC cells *in vivo*. (*a*) Mean volumes of xenografted A549 derived tumors in mice treated with vehicle control (ctrl) or ruxolitinib (Ruxo) at 90 mg/kg body weight, seven times per week, BID, and (*b*) the endpoint tumor weight. (*c*) Representative pictures of A549 tumor‐derived xenografts after ctrl or Ruxo treatment. (*d*) Representative pictures of immunohistochemical stainings for KI67, cleaved caspase 3 (CC3) and p‐STAT3 staining of A549 cell line derived xenograft tumors upon ctrl and Ruxo treatment (scale bar: 100 μm), and (*e*) quantitation of positive cells for respective stainings. (*f*) Relative mRNA expression of human transcript variants of indicated genes normalized to human housekeeping genes (*28S*, *TBP*, *ACTB*) in A549 derived xenografted tumors. (*d*–*f*) *n* = ≥ 5 tumors per group. For all graphs Student's *t*‐test: *p < 0.05, **p < 0.01, ***p < 0.001. For all graphs data presented as means ± SEM. [Color figure can be viewed at http://wileyonlinelibrary.com]

Next, we assessed the potential benefits of pharmacological JAK1/2 inhibition in K‐RAS‐driven lung AC using the JAK1/2 selective tyrosine kinase inhibitor ruxolitinib. We performed subcutaneous engraftments of human lung AC cell lines harboring oncogenic K‐RAS mutations into immunodeficient NOD scid gamma mice. Intriguingly, ruxolitinib treatment significantly attenuated growth of the human *K‐RAS* ^*G12S*^‐mutated lung AC cell line A549 (Figs. 2 *a*–2 *c*), and impaired growth of A427 (*K‐RAS* ^*G12D*^) transplanted cells (Figs. S2 *a* and S2 *b*). Immunohistochemistry analysis of proliferation and apoptosis markers within tumors revealed that JAK inhibition significantly reduced proliferation and slightly increased apoptosis of transplanted lung AC cells (Figs. 2 *d* and 2 *e*). JAK1/2 inhibition in tumor cells of ruxolitinib treated mice was efficient, since activation of the downstream effector STAT3 was almost absent after tyrosine kinase inhibitor treatment (Figs. 2 *d* and 2 *e*). Accordingly, ruxolitinib treatment impaired a well‐described autocrine oncogenic activation loop in this model.^10^ Indeed, tumor cell‐derived expression of pro‐inflammatory cytokines *IL‐6* and *IL‐1β* was significantly decreased in the ruxolitinib treated group, as verified by quantitative real‐time PCR using primers specific for the human transcripts of these cytokines (Fig. 2 *f*). Notably, the ruxolitinib dose used in our experiments was generally well tolerated by the experimental mice, and we did not observe any significant body weight loss in the treated groups (Fig. S2 *c*). Since abrogation of JAK signaling may also impair angiogenesis,^35^ we checked expression of the angiogenic markers *Vegfα* and *Hif1α* and evaluated numbers of cluster of differentiation 31^+^ vessels, but we did not observe changes upon ruxolitinib treatment (Figs. S2 *d* and S2 *e*).

### Ruxolitinib attenuates tumorigenesis of autochthonous *K‐RAS‐*driven lung AC

**Figure 3:**
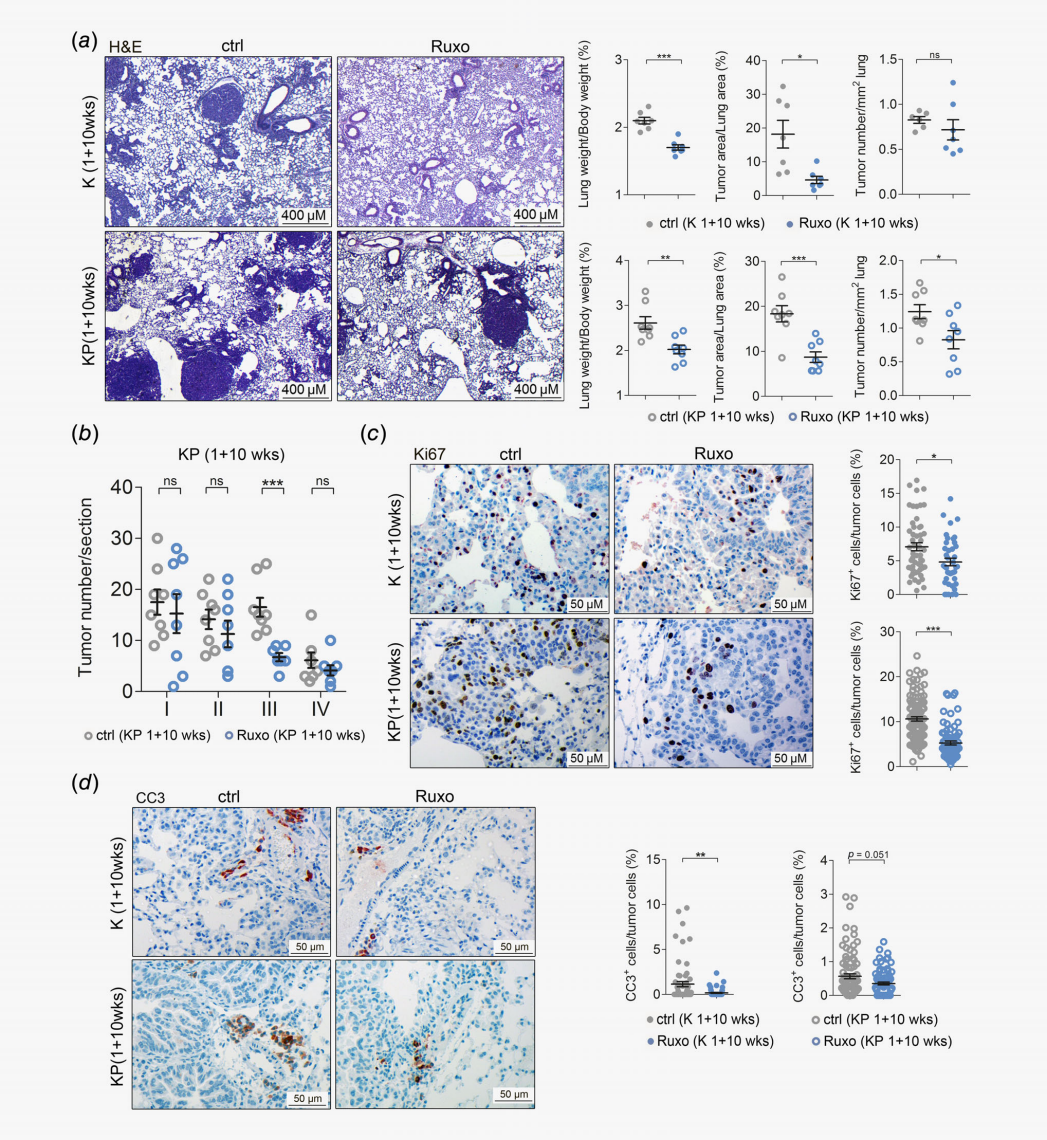
Ruxolitinib attenuates tumorigenesis of autochthonous K‐RAS‐driven lung AC. (*a*) Left panel: representative pictures of hematoxylin and eosin stained lung sections of vehicle control (ctrl) or ruxolitinib (Ruxo) treated *K*‐*ras* ^*G12D*^ (K) mice (*n* = 7 per group) and *K*‐*ras* ^*G12D*^:*p53* ^*fl/fl*^ (KP) mice (*n* = 8 per group). Treatment was started 1 week post tumor initiation, and continued for 10 weeks, with ruxolitinib being administered at 90 mg/kg body weight, seven times per week, BID (scale bars: 400 μm). Right panel: quantitation of hematoxylin and eosin stained lung sections from K mice (upper) and KP‐mice (lower) treated with ctrl or Ruxo started 1 week post tumor initiation and continued for 10 weeks. (*b*) Graph depicts tumor numbers per section stratified by tumor grades. Per mouse, one section was analyzed (*n* = 8 mice per group). (*c*) Left panel: representative pictures of immunohistochemical stainings for KI67 positive cells of lungs stemming from ctrl and Ruxo treated K and KP mice. Right panel: quantitation of indicated stainings. (*d*) Left panel: cleaved caspase‐3 positive cells of lungs of ctrl and Ruxo treated K and KP mice. Right panel: quantitation of indicated stainings (scale bars: 50 μm). (*c*, *d*) Mann–Whitney *U*‐test, *p < 0.05, **p < 0.01, ***p < 0.001. Others: Student's *t*‐test. *p < 0.05, **p < 0.01, ***p < 0.001. For all graphs data presented as means ± SEM. [Color figure can be viewed at http://wileyonlinelibrary.com]

Although cell‐line‐derived xenograft models have the advantage to study tumors of human origin, a major drawback of these models is the absence of a functional immune system.^36^ However, this is a critical factor in our study, since ruxolitinib was reported to promote immunosuppression and could thereby eventually enhance tumor progression.^18^ Thus, we addressed the effects of JAK1/2 inhibition in fully immunocompetent mouse models of autochthonous *K‐RAS*‐driven lung AC. Therefore, we took advantage of the *K‐ras* ^*LSL‐G12D*^ (K) and *K‐ras* ^*LSL‐G12D*^:*p53* ^*fl/fl*^ (KP) mice. Inhalation of K and KP mice with a Cre‐recombinase expressing adenovirus (Ad.Cre) triggers *K‐ras* ^*G12D*^ activation in lung epithelial cells, resulting in formation of p53 expressing (K) or deficient (KP) lung ACs. Of note, KP mice develop lung ACs showing features of advanced grade tumors.^28^, ^37^ First, we validated the mouse models by isolating primary lung alveolar type II cells derived from K mice and inducing the expression of oncogenic KRAS *in vitro*. Intriguingly, global gene expression analysis revealed that *K‐ras* ^*G12D*^ expression resulted in activation of the JAK–STAT pathway (Figs. S3 *a* and S3 *b*).^27^ Encouraged by these data, we treated K and KP mice 1 week after tumor induction with ruxolitinib or vehicle control for a period of 10 weeks, euthanized them and analyzed their lungs. We noticed a significant decrease in tumor burden in ruxolitinib treated mice compared to vehicle‐treated control mice, as indicated by reduced lung weight to body weight and tumor to lung area ratios, concomitant with reduced tumor numbers (Fig. 3 *a*). Of note, sex differences in treatment response were not observed in our mouse models (Fig. S4 *a*). Furthermore, grading of tumors by a pathologist (K.D.) revealed that ruxolitinib blocked the progression of K‐RAS‐driven lung tumors. Indeed, ruxolitinib treatment resulted in a significant reduction of high grade tumors compared to vehicle control, which reached significance for grade III tumors (Fig. 3 *b* and Fig. S4 *b*). No differences in body weights were observed between control and treatment groups (Figs. S4 *c* and S4 *d*). Similar to the engrafted human lung AC cells, ruxolitinib significantly attenuated tumor cell proliferation in these mice (Fig. 3 *c*). Intriguingly, we observed a higher rate of tumor cell apoptosis in the vehicle treated compared to ruxolitinib group in both K and KP mouse models (Fig. 3 *d*). Importantly, there were no differences in the expression levels of the angiogenic factors *Hif1α* and *Vegfα* between control and ruxolitinib treatment groups (Figs. S4 *e* and S4 *f*), suggesting that impairment of tumor cell proliferation is responsible for the reduced tumor burden upon ruxolitinib treatment in these models.

### JAK inhibition impairs progression of established *K‐RAS*‐mutated lung AC and modulates the TME

**Figure 4:**
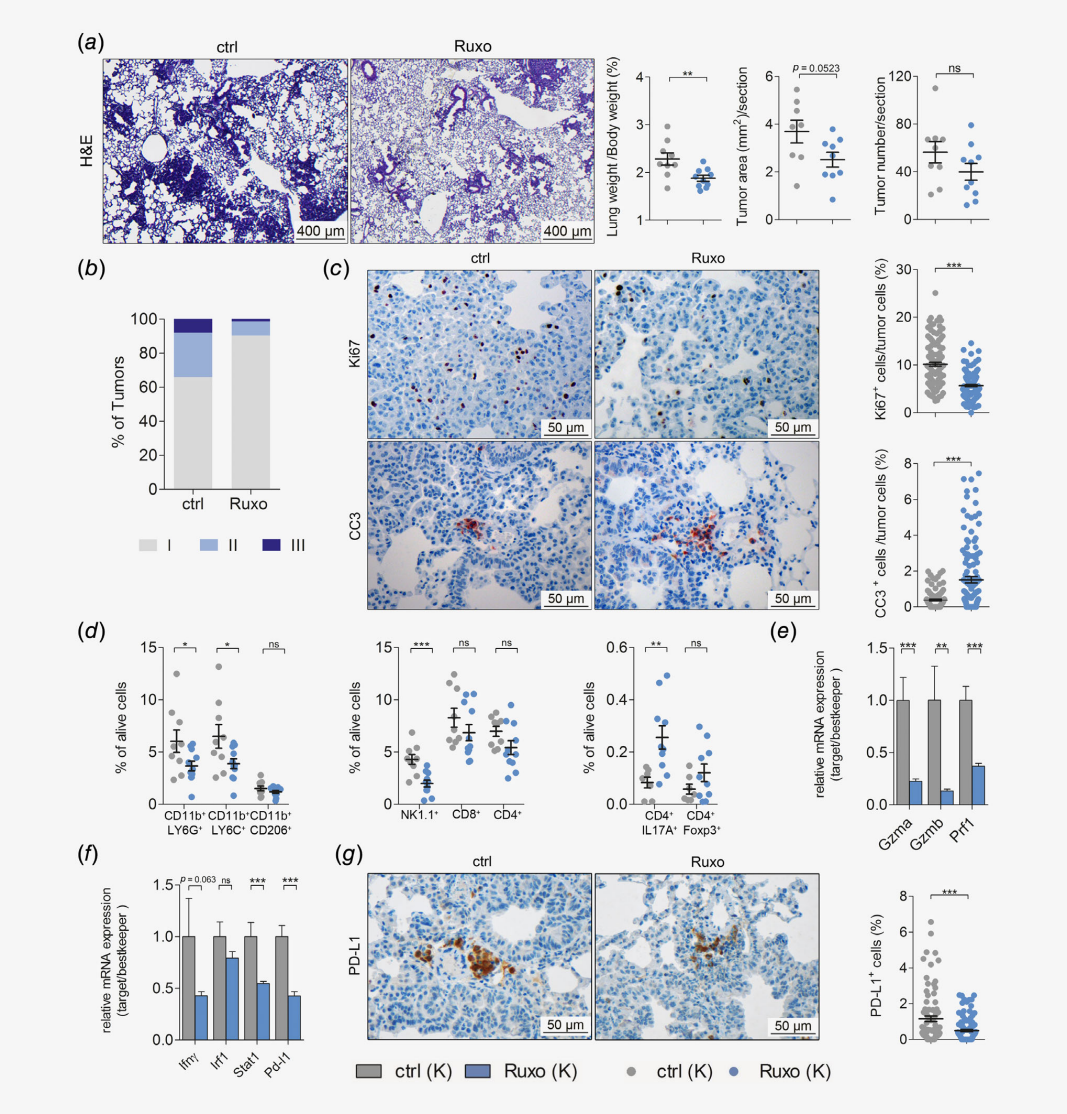
JAK inhibition impairs progression of established K‐RAS‐mutated lung AC and alters TME. (*a*) Left panel: representative pictures of hematoxylin and eosin stained lung sections of vehicle control (ctrl) or ruxolitinib (Ruxo) treated *K*‐*ras* ^*G12D*^ (K) mice. Right panel: quantitation of hematoxylin and eosin stained lung sections of ctrl or Ruxo treated *K*‐*ras* ^*G12D*^ (K) mice. Treatment was started 8 week post tumor initiation, and continued for 5 weeks (8 + 5wks), with ruxolitinib being administered at 90 mg/kg body weight, seven times per week, BID (scale bars: 400 μm) (*n* ≥ 9 per group). (*b*) Graph represents the percentage of tumors with indicated grades in vehicle control and ruxolitinib treated K mice. (*c*) Left panel: representative pictures of immunohistochemical stainings for KI67 and cleaved caspase‐3 positive cells, comparing lungs of ctrl or Ruxo treated K mice (8 + 5 weeks). Right panel: quantitation of indicated stainings (scale bars: 50 μm). (*d*) Results of flow cytometric analysis depicting the percentage of myeloid cells (left), lymphoid cells (middle) and CD4^+^ subsets (right) in tumor‐harboring lung lysates of ctrl and Ruxo treated K mice (8 + 5 weeks). (*e*) Graph displaying relative mRNA expression of the indicated genes normalized to housekeeping genes (*28s*, *Tbp*, *Actb*) in tumor harboring lungs of ctrl *versus* Ruxo treated K mice (8 + 5 weeks). (*f*) Graph indicating relative mRNA expression of the indicated genes normalized to housekeeping genes (*28s*, *Tbp*, *Actb*) in tumor harboring lungs of ctrl *versus* Ruxo treated K mice (8 + 5 weeks). (*g*) Left panel: representative pictures of immunohistochemical staining for PD‐L1 positive cells of lungs stemming from ctrl and Ruxo treated K mice (8 + 5 weeks). Right panel: quantitation of indicated staining. (*c*, *g*) Mann–Whitney *U*‐test. Others: Student's *t*‐test. For all graphs data presented as means ± SEM. *p < 0.05, **p < 0.01, ***p < 0.001. [Color figure can be viewed at http://wileyonlinelibrary.com]

Having demonstrated that ruxolitinib effectively reduces tumorigenesis when administered at early stages of tumor formation, we questioned whether ruxolitinib prevents progression of already established K‐RAS‐driven lung AC in a therapeutic setting. Therefore, we treated K mice with ruxolitinib starting 8 weeks post Ad.Cre inhalation for five consecutive weeks. In this model, we observed a reduction in lung to body weight ratios and total tumor area per lung section after ruxolitinib treatment as compared to vehicle control treatment, while tumor numbers remained unaltered (Fig. 4 *a*). Notably, analysis of tumor to lung area ratios did not show differences between the treatment groups, most likely because total lung areas were smaller upon ruxolitinib treatment (Figs. S5 *a*–S5 *c*). Strikingly, grading of tumors revealed a reduction of high‐grade tumors, namely tumors of grades II and III (Fig. 4 *b*). In addition, ruxolitinib treatment of mice with established tumors also triggered significant reduction in tumor cell proliferation and increased apoptosis (Fig. 4 *c*). Similar to the previous models, differences in mRNA expression levels of angiogenic markers *Hif1α* and *Vegfα* were not observed and body weights showed no significant differences between control and ruxolitinib treatment groups (Figs. S5 *d* and S5 *e*). These data suggests that ruxolitinib impairs the progression of established K‐RAS‐driven lung AC. Since JAK signaling plays a crucial role in regulating the immune system, we analyzed if JAK1/2 inhibition affected the composition of the TME by performing flow cytometric analysis of lung tumors.^17^ Indeed, the abundance of tumor promoting granulocytic CD45^+^CD11b^+^LY6G^+^ and monocytic CD45^+^CD11b^+^LY6C^+^ MDSC^38^ was significantly reduced in the lungs of tumor‐bearing, ruxolitinib‐treated mice. Furthermore, the number of tumor promoting CD45^+^CD11b^+^CD206^+^ M2 macrophages was lowered upon JAK1/2 inhibition (Fig. 4 *d*). With respect to the lymphoid lineage, our analysis revealed significantly diminished NK cell numbers upon ruxolitinib administration in tumor bearing lungs of these mice (Fig. 4 *d*). These data are in line with a previous report using a preclinical breast cancer model.^18^ In addition, we observed a slight reduction in infiltrating CD8^+^ and CD4^+^ T cells upon ruxolitinib treatment, but an increase in CD4^+^IL17A^+^ Th17 cells as well as CD4^+^Foxp3^+^ regulatory T cells (Fig. 4 *d*). Moreover, JAK inhibition lead to a significant impairment of NK and CD8^+^ T cell activation, but on the other hand also abrogated interferon‐γ‐mediated STAT1 signaling and hence reduced PD‐L1 expression (Figs. 4 *e*–4 *g*). In summary, these data indicated that ruxolitinib altered the myeloid compartment of the TME toward an antitumorigenic state, which out‐competed the suppressing effects of ruxolitinib on infiltrating cytotoxic NK and CD8^+^T cells.

### JAK inhibition abrogates expression of oncogenic chemokines and cytokines

**Figure 5:**
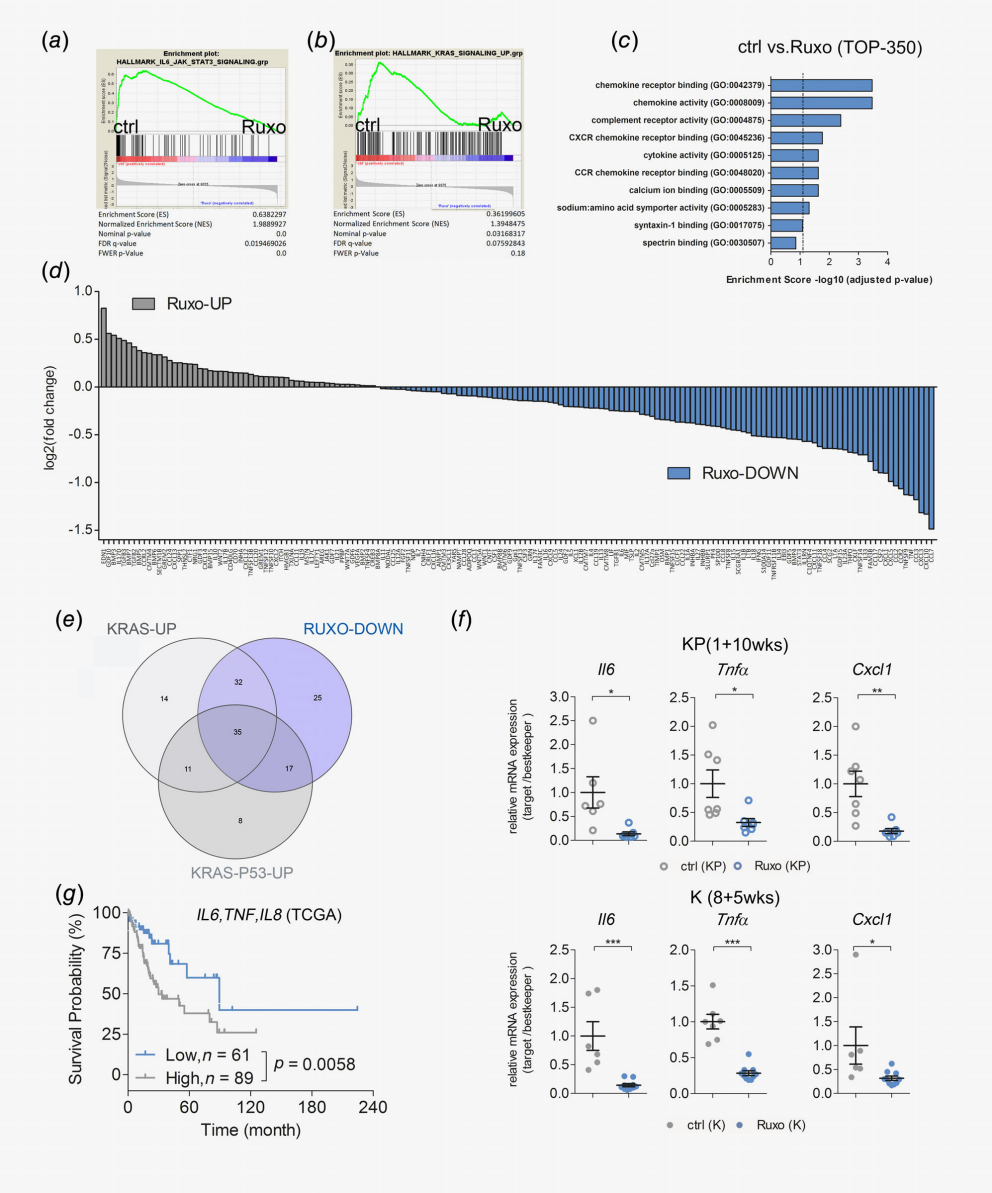
JAK inhibition abrogates expression of oncogenic chemokines and cytokines. (*a*) GSEA of RNA‐seq data of lungs of *K*‐*ras* ^*G12D*^ (K) mice 8 weeks post tumor induction, treated with vehicle control (ctrl) or ruxolitinib (Ruxo) for four consecutive days before harvesting (*n* = 4 per group). The used genesets are HALLMARK_IL6_STAT3_SIGNALING and (*b*) HALLMARK_KRAS_SIGNALING_UP, comparing ctrl and Ruxo treatment. (*c*) Graph depicting the adjusted p‐values of enrichment scores of indicated GO‐molecular function pathways in ctrl *versus* Ruxo treated K mice. The top 350 genes downregulated in lungs of Ruxo treated mice compared to ctrl treated mice were included in the analysis. (*d*) Graph shows DESeq calculated log2 fold changes in mRNA expression of indicated up‐ or downregulated chemokine/cytokine related genes in tumor bearing lungs of Ruxo treated compared to ctrl treated K mice. (*e*) Venn diagram depicting amount of overlapping chemokine and cytokine related genes in all three groups. The defined 35 common genes were found by intersecting the after gene data sets: genes downregulated in tumor bearing lungs of Ruxo treated mice compared to ctrl treated mice (RUXO‐DOWN), genes upregulated in *K‐ras* ^*G12D*^ mutated alveolar type‐II cells compared to WT alveolar type‐II cells (KRAS‐UP, GSE113146) and genes upregulated in *K‐ras‐p53‐*mutated tumor‐harboring lungs compared to WT lungs from KP mice (KRAS‐P53‐UP). (*f*) Relative mRNA expression of the indicated genes normalized to housekeeping genes (*28s*, *Tbp*, *Actb*) in tumor harboring lungs of vehicle control *versus* ruxolitinib treated KP mice (upper) and K mice (lower). (*g*) Graph depicts prognostic value of indicated genes in human *K‐RAS*‐mutated lung AC samples. Patients were stratified according to auto best cut‐off selection. Data were retrieved from the Cancer Genome Atlas (*n* = 150). Log‐rank test was used for statistical analysis. For (*f*) Student's *t*‐test. *p < 0.05, **p < 0.01, ***p < 0.001. Data presented as means ± SEM. [Color figure can be viewed at http://wileyonlinelibrary.com]

Next, we thought to unravel the transcriptional changes, leading to the establishment of an antitumorigenic TME upon ruxolitinib treatment. Therefore, we performed RNA‐seq analysis of short‐term (4 days) treated tumor‐bearing lungs from K mice with already established lung ACs. As expected, short‐term administration of ruxolitinib significantly diminished activation of JAK–STAT signaling in the lung lysates of these mice (Fig. 5 *a* and Fig. S6 *a*). Intriguingly, GSEA analysis also revealed that ruxolitinib administration significantly reduced K‐RAS activity in these lungs (Fig. 5 *b*). Notably, within the top 350 genes negatively regulated by ruxolitinib (Table S6), we noticed an over‐representation of factors found within GO clusters for chemokine and cytokine activity (Fig. 5 *c* and Fig. S6 *b*). Accordingly, most of the genes annotated in these gene sets were downregulated upon ruxolitinib treatment compared to vehicle control (Fig. 5 *d* and Table S6). Importantly, 84 of these 109 genes downregulated by ruxolitinib were either upregulated in *K‐ras* ^*G12D*^‐mutated lung alveolar type II (K) cells compared to WT cells (67 genes) or in tumor harboring lungs of KP mice compared to healthy WT lungs (52 genes). Notably, 35 hits (Tables S2 and S6) were common in all three groups (Fig. 5 *e* and Figs. S6 *c* and S6 *d*). Among these 35 genes, key pro‐inflammatory and pro‐tumorigenic factors including *ll6*, *Tnfα* and chemokine (C‐X‐C motif) ligand 1 (*Cxcl1*) were found. The anti‐inflammatory properties of ruxolitinib were also observed in the long‐term treatment models, as evidenced by decreased expression of these genes in treated K and KP mice (Fig. 5 *f*).

Next, we evaluated the implication of these chemo/cytokines for human lung AC pathogenesis. Therefore, we took advantage of a meta‐analysis based tool for the assessment of potential biomarkers in lung AC.^25^ Multigene classifier analysis revealed that lower mRNA expression of the signature comprising of 35 genes was associated with a better survival prognosis in these patients at a false discover rate < 1% (Fig. S6 *e* and Table S2). Moreover, lower mRNA levels of *lL6*, *TNFα* and *IL8*, the human orthologue to mouse *Cxcl1* (Fig. 5 *g*), could be associated with a better prognosis in lung AC patients harboring *K‐RAS* mutations.^39^ Taken together, we provide here evidence that ruxolitinib treatment succesfully impairs K‐RAS activity in lung tumors and leads to the establishment of an anti‐inflammatory and antitumorgenic tumor microenviroment.

## Discussion

Being a well‐defined culprit in myeloproliferative disorders, the JAK/STAT pathway is a well‐known target for pharmaceutical industry. Ruxolitinib was already Food and Drug Administration approved in 2011 based on the positive results of the COMFORT‐I clinical trial.^40^ Somatic mutations rendering JAK2 constitutively active like the V617F mutation are often found in hematologic malignancies. However, solid cancers including lung cancer lack such activating somatic mutations.^23^, ^41^ Nevertheless, clinical trials for lung AC patients were launched using JAK inhibitors ruxolitinib, in particular for patients with activating *EGFR*‐mutations upstream of JAK1 and JAK2 (NCT02155465, NCT02145637, NCT02917993, NCT03450330). A recent clinical trial specifically excluding patients with *EGFR*‐mutations showed a longer overall survival (5.9 *vs*. 7.5 months for placebo treated *vs*. ruxolitinib treated, hazard ratio = 0.877) when combining ruxolitinib and chemotherapy in non‐small cell lung cancer patients (NCT02119650). However, the *K‐RAS* mutation status of the involved patients was not disclosed. Therefore the potential benefit of a JAK1/2 inhibition for K‐RAS‐driven lung AC pathogenesis cannot be extrapolated. Hence, as in other clinical trials when using for example mitogen‐activated protein kinase kinase 1 or rapidly accelerated fibrosarcoma inhibitors, it would be important for JAK1/2 targeting clinical trials to stratify for the *K‐RAS* mutational status.

Here, we found increased expression of JAKs and activation of downstream STAT signaling in patients harboring advanced K‐RAS lung AC. Increased JAK levels also correlated with enhanced K‐RAS activity in human lung AC patient samples, suggesting that JAK/STAT signaling fuels the proliferation of *K‐RAS‐*mutated lung AC. Accordingly, ruxolitinib treatment markedly reduced tumor cell proliferation of human *K‐RAS*‐mutated A549 cells engrafted in immunodeficient NOD scid gamma mice and reduced a K‐RAS activation gene signature. The engrafted tumors showed decreased expression of tumor cell‐derived, pro‐oncogenic cytokines *IL‐1β* and *IL‐6*, suggesting that ruxolitinib interferes with feed‐forward cytokine signaling which promotes K‐RAS tumorigenesis.^10^ Furthermore, ruxolitinib treatment resulted in tumor growth reduction in immunocompetent experimental mouse models. Ruxolitinib impaired K‐RAS‐driven lung AC not only at early tumor stages but also in established tumors, independently of the p53 status. Early treatment of tumors with ruxolitinib resulted in reduced tumor cell proliferation and reduced apoptosis levels. By contrast, we observed reduced cell proliferation and increased apoptosis rates in established tumors upon ruxolitinib treatment. This discrepancy can be interpreted as the result of a higher cell turnover in early tumorigenesis or by a tumor‐compensatory mechanism upon JAK1/2 inhibition present at early tumor stages. In agreement with previous reports, ruxolitinib treatment diminished tumor infiltrating CD8^+^ and CD4^+^ T cells and significantly lowered NK cell numbers but did not result in tumor metastasis, even in the absence of p53.^17^, ^18^, ^42^ In agreement with others, JAK inhibition abrogated interferon‐γ‐mediated STAT1 signaling and reduced PD‐L1 expression levels.^43^ Nevertheless, NK and CD8^+^ T cell activation markers were reduced in lungs of ruxolitinib treated mice. Furthermore, ruxolitinib elevated numbers of tumor infiltrating CD4^+^IL17A^+^ Th17 and CD4^+^Foxp3^+^ regulatory T cells. Of note, T cells potentially play a minor role in our experimental mouse models due to a lack of tumor immunogenicity. In this sense, assessment of JAK inhibitors in T‐cell‐dependent lung AC experimental models^44^ may be a better option and can be more informative to design and interpret future and ongoing clinical trials, especially those combining JAK inhibitors and immune checkpoint blockers (e.g., NCT03425006).

On the other hand, systemic JAK inhibition triggered a slight reduction of tumor promoting M2 macrophages and significantly reduced the abundance of infiltrating granulocytic and monocytic MDSCs, which is in agreement with an induction of an antitumorigenic TME and the antitumor effects of ruxolitinib. Importantly, MDSCs and M2 tumor‐associated macrophages not only impede tumor immune surveillance but also support tumor cell proliferation *via* cytokine production, and could thereby foster growth of K‐RAS‐driven lung AC.^38^, ^45^ In line with these data, ruxolitinib treatment of mouse autochthonous lung tumors mainly affected the expression of pro‐tumorigenic chemokines and cytokines. This chemokine/cytokine milieu is well‐known to be a central modulator of the cancer microenvironment and it has been established as one of the hallmark drivers of cancer.^46^ Among these prominently downregulated chemokines and cytokines upon ruxolitinib treatment, we found *ll6*, *Tnfα* and *Cxcl1*. All of them are well‐described pro‐inflammatory and pro‐tumorigenic chemo‐ and cytokines. Reduced IL6 levels were shown to trigger a switch in the TME toward an antitumoral phenotype.^47^ TNFα directly endorses tumor progression and metastasis.^48^ Furthermore, CXCL1 has been reported to facilitate lung cancer cell growth due to the recruitment of MDSCs and tumor associated neutrophils.^13^, ^49^ Importantly, all of these factors were also found to be upregulated in *K‐ras* ^*G12D*^‐mutated lung alveolar type II (K) cells compared to WT cells or in tumor harboring lungs from KP mice compared to non‐tumorous lungs from KP mice. In agreement with this data, low mRNA expression levels of *lL6*, *Tnfα* and *IL8* (the human orthologue to murine *Cxcl1*) were also associated with a better prognosis in lung AC patients harboring *K‐RAS* mutations.^39^ Thus, these data indicate that *lL6*, *Tnfα* and *Cxcl1* play a central role for K‐RAS driven lung AC both in human and mice and provides a solid rationality for using agents blocking inflammation‐driven tumorigenesis, such as ruxolitinib, to treat lung AC.^7^

In summary, we demonstrated here that JAK‐mediated signaling is involved in progression of K‐RAS‐driven lung tumorigenesis and that pharmacological inhibition of JAK/STAT signaling blocks the cytokine‐mediated feed‐forward loop responsible for tumor cell proliferation. Additionally, JAK inhibition reprograms the TME toward an anti‐inflammatory/antitumorigenic state. Therefore, our data suggest that ruxolitinib treatment represents an attractive therapeutic opportunity for treatment of *K‐RAS*‐mutated lung AC.

## Supporting information


**Fig. S1**: JAK mediated signaling is activated in progressing K‐RAS‐mutated human lung AC.
**Figure S2**: JAK inhibition impairs human K‐RAS‐mutated lung AC cell growth *in vivo*.
**Figure S3**: K‐RAS^G12D^ transformed primary pneumocytes exhibit JAK–STAT pathway activation *in vitro*
**Figure S4**: Ruxolitinib attenuates tumorigenesis of autochthonous K‐RAS‐driven lung AC
**Figure S5**: JAK inhibition impairs progression of established K‐RAS‐mutated lung AC
**Figure S6**: JAK inhibition abrogates expression of oncogenic chemokines and cytokines

**Table S1**. Probeset‐IDs‐GSE75037
**Table S2**. Probeset‐IDs‐Cytokines‐Chemokines
**Table S3**. Genotyping primers
**Table S4**. Quantitative PCR analysis primers
**Table S5**. Antibodies for Flow Cytometric analysis

**Table S6** Chemokine & cytokine gene names

## Data Availability

All materials will be made available to the scientific community. RNAseq data are available within the sequence read archive (SRA) with the accession number PRJNA518030.
