# Supplementary material for: JAK–STAT inhibition impairs K‐RAS‐driven lung adenocarcinoma progression
Source: Int J Cancer. 2019 Sep 10;145(12):3376–88. doi: 10.1002/ijc.32624 (PMC6856680; doi:10.1002/ijc.32624)
Supplement: Supplementary file 1 — Fig. S1: JAK mediated signaling is activated in progressing K‐RAS‐mutated human lung AC. Figure S2: JAK inhibition impairs human K‐RAS‐mutated lung AC cell growth in vivo. Figure S3: K‐RASG12D transformed primary pneumocytes exhibit JAK–STAT pathway activation in vitro Figure S4: Ruxolitinib attenuates tumorigenesis of autochthonous K‐RAS‐driven lung AC Figure S5: JAK inhibition impairs progression of established K‐RAS‐mutated lung AC Figure S6: JAK inhibition abrogates expression of oncogenic chemokines and cytokines [file IJC-145-3376-s001.docx]

**Supplementary Figures**


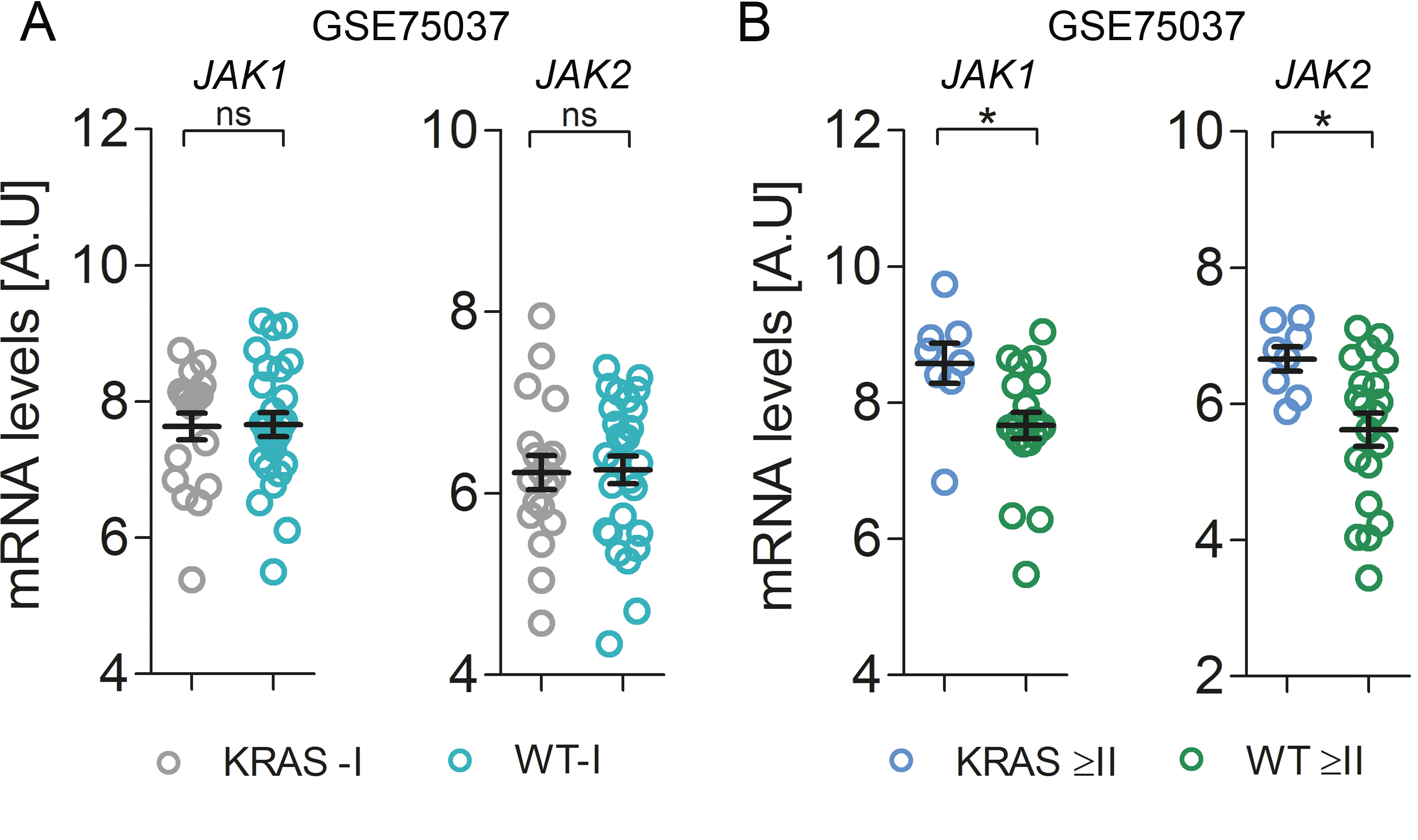


*Fig.S1: JAK mediated signaling is activated in progressing K-RAS-mutated human lung AC.*

**A.)** Graph showing relative *JAK1* (left) or *JAK2* (right) mRNA expression levels comparing human lung AC tissue of stage I with (KRAS-I, n=19) or without (WT-I, n=28) *K-RAS*-mutation. **B.)** Graph showing relative *JAK1* (left) or *JAK2* (right) mRNA expression levels comparing human lung AC tissue of stage II or higher with (KRAS≥II, n=8) or without (WT≥II, n= 20) *K-RAS* mutation. Data represent mean ± S.E.M., A.U (arbitrary units), Student’s *t*-test: *p<0.05.Data in A) and B) were retrieved from the Gene Expression Omnibus (GSE75037).

*Fig.S2:* *JAK inhibition impairs human K-RAS-mutated lung AC cell growth in vivo.*


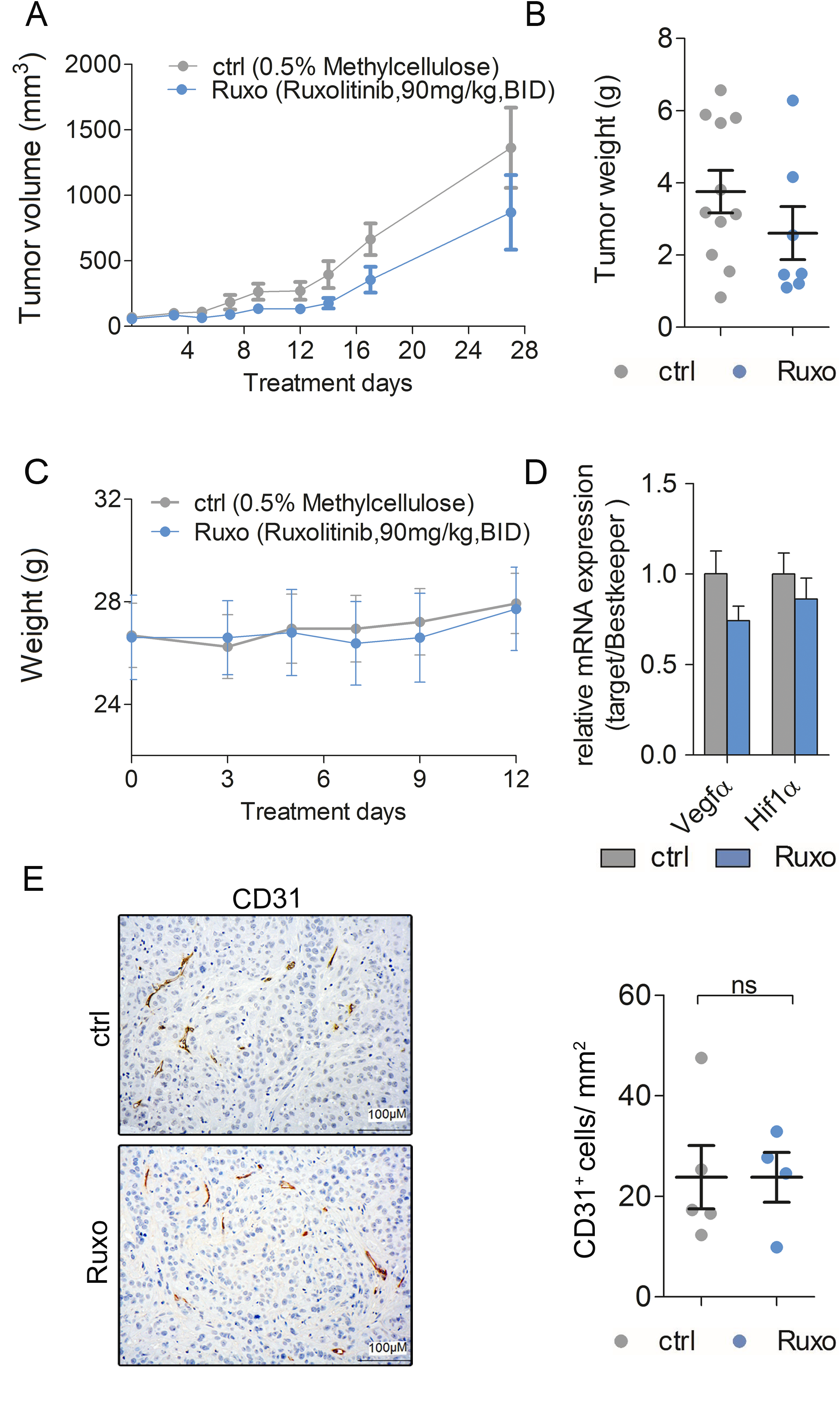


**A)** Mean volumes ± SEM. of xenografted A427 derived tumors in mice treated with vehicle control (ctrl) or ruxolitinib (Ruxo) at 90 mg/kg body weight, 7 times per week, BID, and **B)** the endpoint tumor weight ± SEM. **C)** Graph showing bodyweight of NSG mice engrafted with A549 cells treated with either ctrl or Ruxo. **D)** Graph displaying relative mRNA expression of mouse variants of selected genes normalized to mouse housekeeping genes (*28s*, *Tbp, Actb*) in tumor isolates. **E)** Representative pictures showing CD31 staining of A549 cell line derived xenografts tumors upon ctrl and Ruxo treatment and quantitation of positive cells for respective staining (Scale bars:100µm). Data presented as means ± S.E.M. Student’s *t*-test.

*Fig.S3:* K-RAS^G12D^ *transformed primary pneumocytes exhibit JAK-STAT pathway activation in vitro*


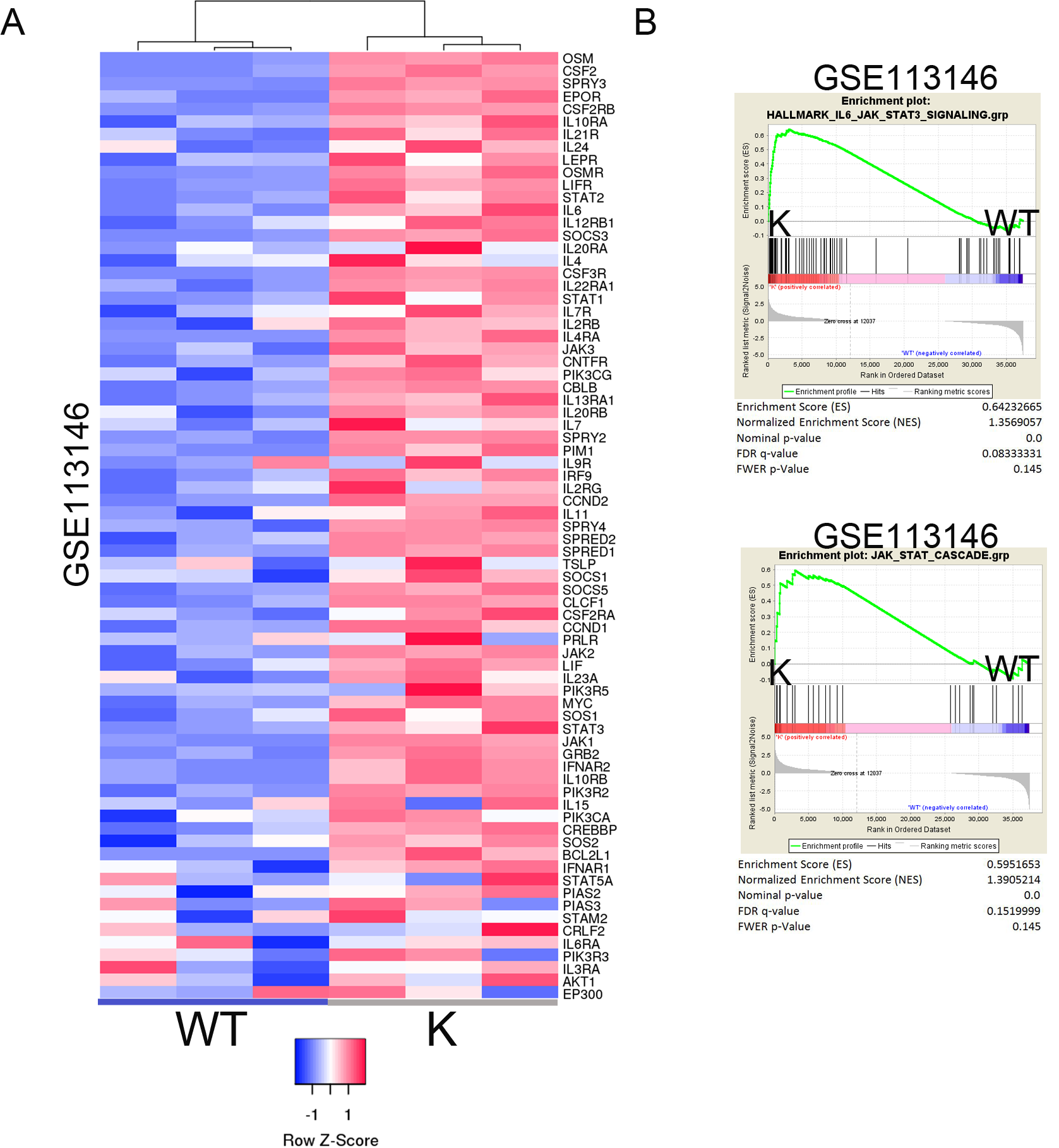


**A)** Heatmap depicting mRNA expression of annotated KEGG-JAK-STAT pathway genes comparing wildtype alveolar type-II cells (WT) versus *K-ras^G12D^* activated alveolar type-II (K) cells isolated from lungs of wildtype and *K-ras^LSL-G12D^* (K) mice. **B)** GSEA for indicated gene sets comparing mouse wildtype alveolar type-II cells (WT) versus *K-ras^G12D^* activated alveolar type-II cells (K). Hierarchical clustering was performed using the heatmapper.ca. online tool. For A) & B) gene expression data were retrieved from GSE113146.

*Fig.S4:* *Ruxolitinib attenuates tumorigenesis of autochthonous K-RAS-driven lung AC*


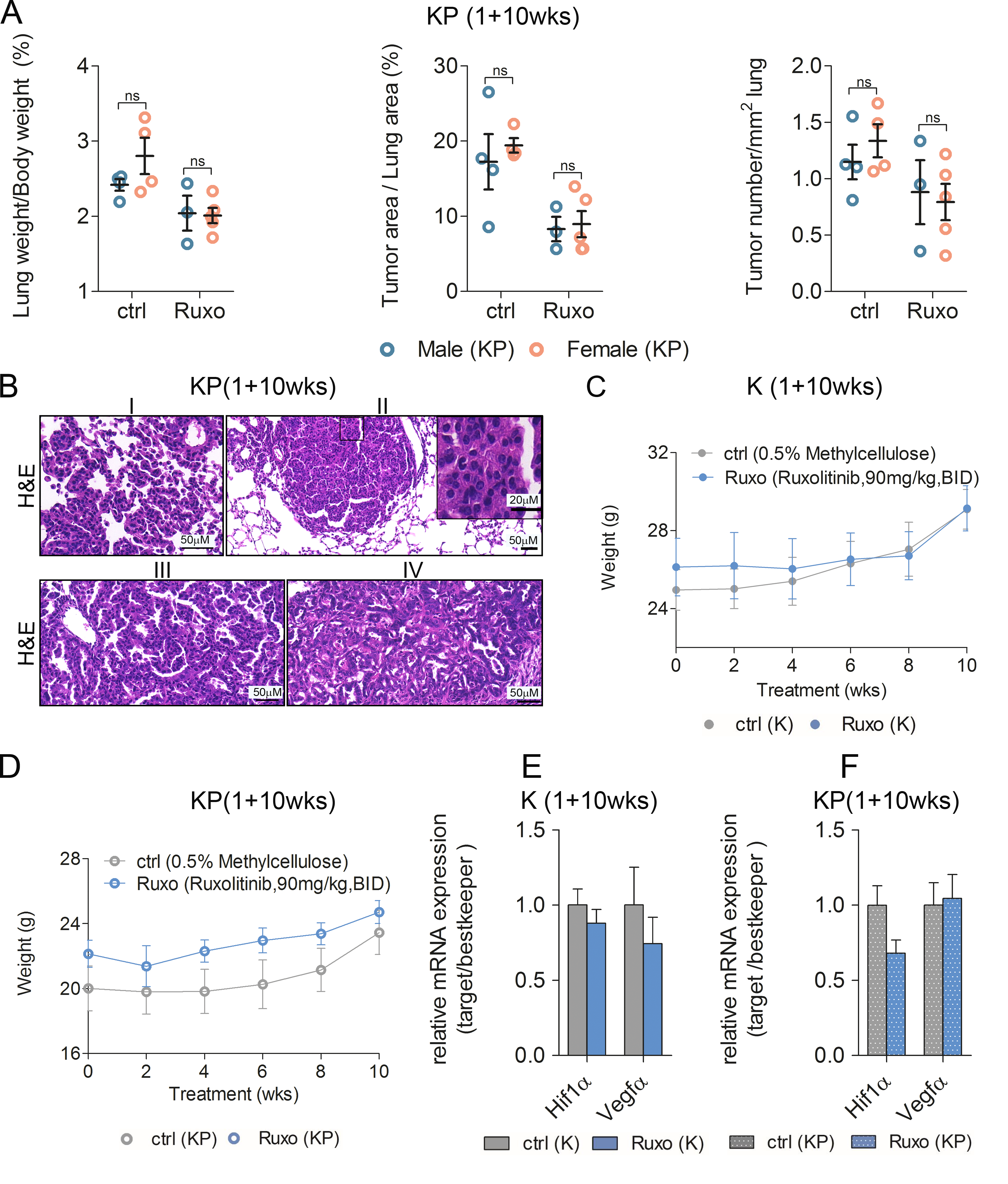


**A)** Quantitation of H&E stained lung sections from *K-ras^G12D^:p53^fl/fl^* (KP-mice) treated with vehicle control (ctrl) or ruxolitinib (Ruxo) started 1 week post tumor initiation and continued for 10 weeks (1+10wks). Graphs depict lung/bodyweight (left), tumor area/lung area (middle) and tumor number per mm^2^ lung (right) stratified into male and female mice**. B)** Representative images of lung tumors with indicated grades. **C)** Graph depicting bodyweight of ctrl and Ruxo treated K mice over the course of the experiment**. D)** Graph depicting bodyweight of ctrl and Ruxo treated KP mice over the course of the experiment. **E)** Relative mRNA expression of indicated genes normalized to mouse housekeeping genes (*28s*, *Tbp,Actb*) in lungs of ctrl and Ruxo treated K mice and **F)** KP mice. Scale bars: 50µm or 20 µm). Data presented as means ± S.E.M. For A) One-way analysis of variance (ANOVA) and Tukey’s multiple comparison test. For others Student’s *t*-test.

*Fig. S5: JAK inhibition impairs progression of established K-RAS-mutated lung AC*


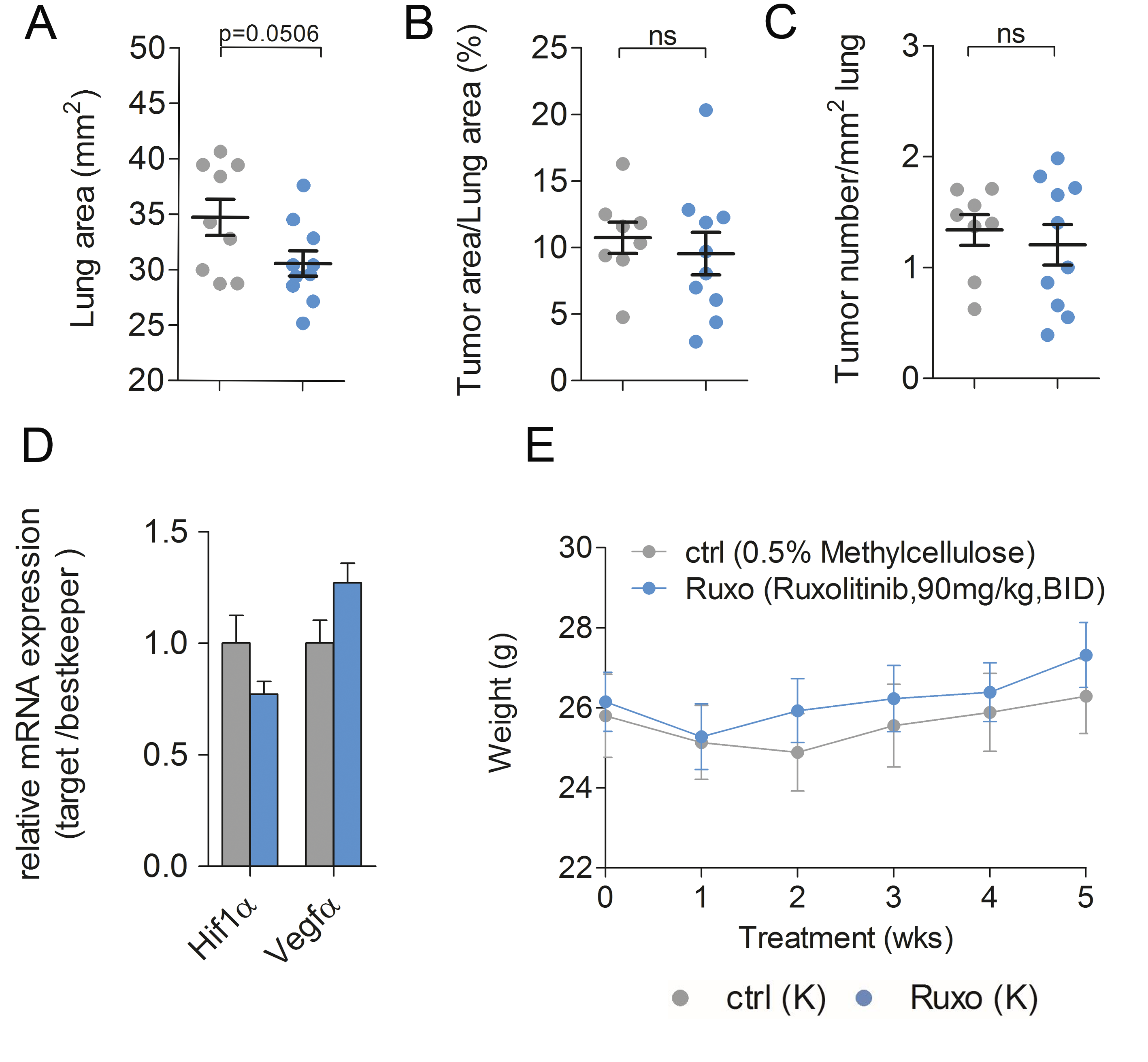


**A)** Blots depicting total lung area, **B)** tumor area to lung area ratios and **C)** tumor numbers per mm^2^ in lungs of K mice treated with vehicle control (ctrl) or ruxolitinib (ruxo). Treatment was started 8 week post tumor initiation and continued for 5 weeks (90 mg/kg body weight, 7 times per wk, BID). **D)** Relative mRNA expression of indicated genes normalized to mouse housekeeping genes (*28s, Tbp, Actb*) in lungs of ctrl and Ruxo treated K mice. **E)** Graph depicting bodyweight of ctrl or Ruxo treated K mice over the course of the experiment. Data presented as means ± S.E.M. Student’s *t*-test.

*Fig.S6: JAK inhibition abrogates expression of oncogenic chemokines and cytokines*


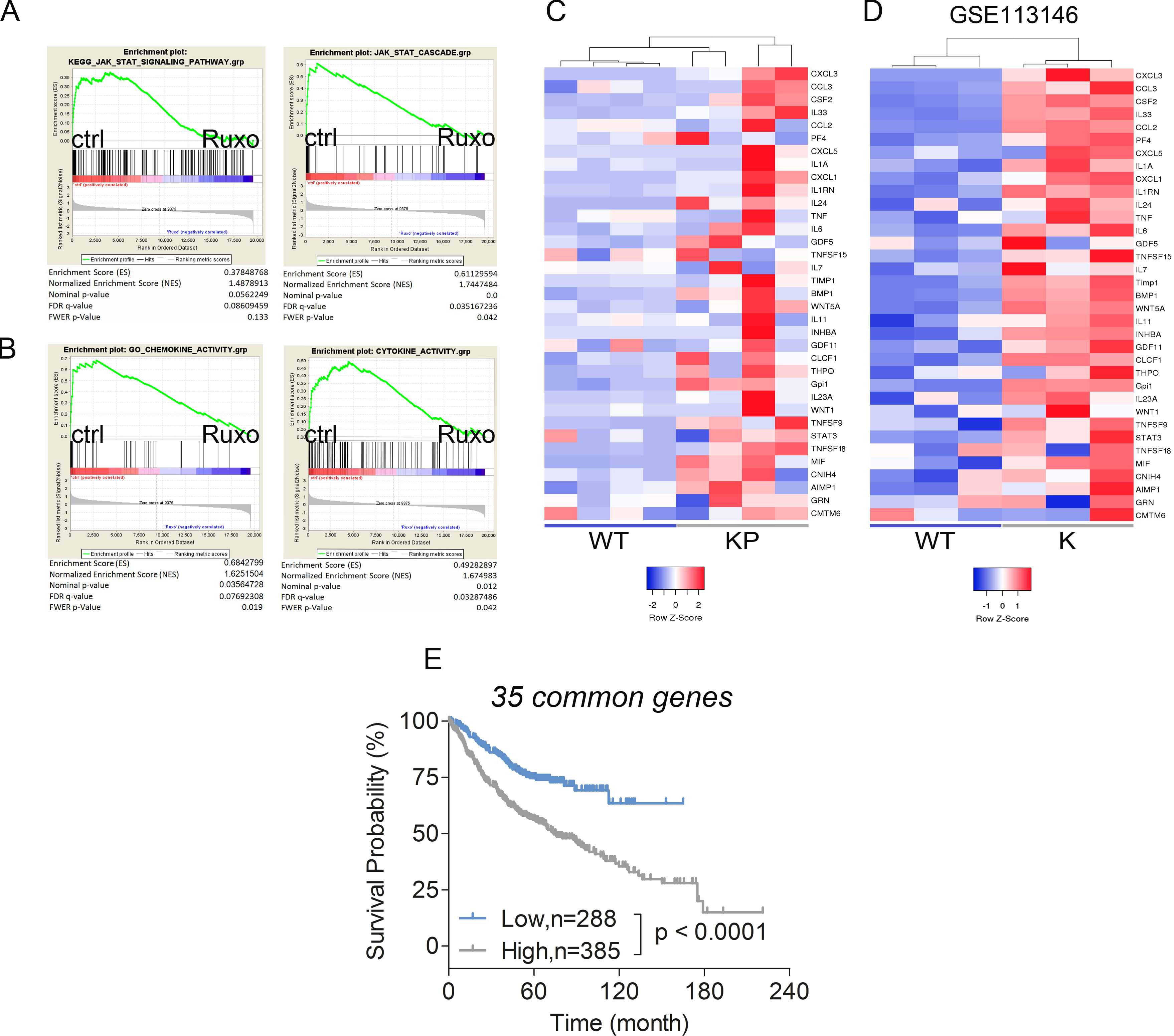


**A)-B)** GSEA for indicated gene sets in lungs of *K-ras^LSL-G12D^* (K) mice, treated for 4 consecutive days with vehicle control (ctrl) or ruxolitinib (Ruxo) starting 8 weeks after tumor induction. **C**) Heatmap depicting mRNA expression of the 35 cytokine and chemokine related hits genes upregulated in tumor harboring lungs of *K-ras^G12D^:p53^∆lep/∆lep^* (KP) mice compared to healthy lungs in wildtype (WT) mice, and **D)** in *K-ras^G12D^* activated alveolar type-II (K) cells compared to wildtype alveolar type-II cells (WT), but downregulated in tumor bearing lungs of Ruxo treated mice compared to ctrl treated mice. Hierarchical clustering was performed using the heatmapper.ca.online tool.**E)** Graph depicts prognostic value of the 35 hits in human lung AC samples. Patients were stratified according to auto best cut-off selection. Data were retrieved used the KM-Plotter online tool. Log-rank test was used for statistical analysis. For D.) Gene expression data were retrieved from GSE113146.
