# Supplementary material for: JAK–STAT inhibition impairs K‐RAS‐driven lung adenocarcinoma progression
Source: Int J Cancer. 2019 Sep 10;145(12):3376–88. doi: 10.1002/ijc.32624 (PMC6856680; doi:10.1002/ijc.32624)
Supplement: Supplementary file 2 — Table S1. Probeset‐IDs‐GSE75037 Table S2. Probeset‐IDs‐Cytokines‐Chemokines Table S3. Genotyping primers Table S4. Quantitative PCR analysis primers Table S5. Antibodies for Flow Cytometric analysis [file IJC-145-3376-s002.doc]

**Table.S1.Probeset-IDs-GSE75037**

| **Gene** | **Probeset-IDs** | **Analysis** |
| --- | --- | --- |
| *JAK1* | ILMN_1793384 | Microarray data (GSE75037) |
| *JAK2* | ILMN_1683178 | Microarray data (GSE75037) |

**Table.S2.Probeset-IDs-Cytokines-Chemokines**

| **Gene** | **Probeset-IDs** | **Analysis** |
| --- | --- | --- |
| CCL3 | 205114_s_at | KM-Plotter-Lung AC |
| CSF2 | 210229_s_at | KM-Plotter-Lung AC |
| IL33 | 209821_at | KM-Plotter-Lung AC |
| CCL2 | 216598_s_at | KM-Plotter-Lung AC |
| PF4 | 206390_x_at | KM-Plotter-Lung AC |
| CXCL5 | 214974_x_at | KM-Plotter-Lung AC |
| IL1A | 210118_s_at | KM-Plotter-Lung AC |
| IL8 | 202859_x_at | KM-Plotter-Lung AC |
| CXCL3 | [207850_at](http://kmplot.com/analysis/index.php?p=service&cancer=lung) | KM-Plotter-Lung AC |
| IL1RN | 212657_s_at | KM-Plotter-Lung AC |
| IL24 | 206569_at | KM-Plotter-Lung AC |
| TNF | 207113_s_at | KM-Plotter-Lung AC |
| IL6 | 205207_at | KM-Plotter-Lung AC |
| GDF5 | 206614_at | KM-Plotter-Lung AC |
| TNFSF15 | 229242_at | KM-Plotter-Lung AC |
| IL7 | 206693_at | KM-Plotter-Lung AC |
| TIMP1 | 201666_at | KM-Plotter-Lung AC |
| BMP1 | 202701_at | KM-Plotter-Lung AC |
| WNT5A | 213425_at | KM-Plotter-Lung AC |
| IL11 | 206924_at | KM-Plotter-Lung AC |
| INHBA | 204926_at | KM-Plotter-Lung AC |
| GDF11 | 226232_at | KM-Plotter-Lung AC |
| CLCF1 | 219500_at | KM-Plotter-Lung AC |
| THPO | 211154_at | KM-Plotter-Lung AC |
| Gpi1 | 204144_s_at | KM-Plotter-Lung AC |
| IL23A | 220054_at | KM-Plotter-Lung AC |
| WNT1 | 208570_at | KM-Plotter-Lung AC |
| TNFSF9 | 206907_at | KM-Plotter-Lung AC |
| STAT3 | 225289_at | KM-Plotter-Lung AC |
| TNFSF18 | 221371_at | KM-Plotter-Lung AC |
| MIF | 203535_at | KM-Plotter-Lung AC |
| CNIH4 | 228437_at | KM-Plotter-Lung AC |
| AIMP1 | 227605_at | KM-Plotter-Lung AC |
| GRN | 211284_s_at | KM-Plotter-Lung AC |
| CMTM6 | 223047_at | KM-Plotter-Lung AC |

**Table.S3.Genotyping primers**

| **Conditional gene** | **Name** | **Sequence** |
| --- | --- | --- |
| *K-ras* | P1 (5'-3') | GTCTTTCCCCAGCACAGTGC |
|  | P2 (5'-3') | CTCTTGCCTACGCCACCAGCTC |
|  | P3 (5'-3') | AGCTAGCCACCATGGCTTGAGTAAGTCTGCA |
| *p53* | P1 (5'-3') | CACAAAAACAGGTTAAACCCAG |
|  | P2 (5'-3') | AGCACATAGGAGGCAGAGAC |

**Table.S4.Quantitative PCR analysis primers**

| **Target** | **Sequence fwd primer (5'-3')** | **Sequence rev primer (5'-3')** |
| --- | --- | --- |
| human *ACTB* | GCACAGAGCCTCGCCTTTGCC | CATGCCCACCATCACGCCCTGG |
| human *28S* | CAGTTCTCTTGGGAATCCAG | TTCAGCAAAGGAGTCAATCCAC |
| human *TBP* | ACGAACCACGGCACTGATTT | GCCAGTCTGGACTGTTCTTCA |
| human *IL1β* | TGAGCTCGCCAGTGAAATGA | CATGGCCACAACAACTGACG |
| human *IL6* | CACAGACAGCCACTCACCTCT | CCTCTTTGCTGCTTTCACACAT |
| mouse *Actb* | CCTGAACCCTAAGGCCAACCG | GCTCATAGCTCTTCTCCAGGG |
| mouse *28s* | ATACCGGCACGAGACCGATAGTCA | GCGGACCCCACCCGTTTACCTC |
| mouse *Tbp* | AGCTCTGGAATTGTACCGCA | AATCAACGCAGTTGTCCGTG |
| mouse *Il6* | TGATGCACTTGCAGAAAACA | ACCAGAGGAAATTTTCAATAGGC |
| mouse *Ifnγ* | GCGTCATTGAATCACACCTG | GAGCTCATTGAATGCTTGGC |
| mouse *Irf1* | GCAAAACCAAGAGGAAGCTG | CAGAGAGACTGCTGCTGACG |
| mouse *Stat1* | GCTGCCTATGATGTCTCGTTT | TGGACATCTGTACGGGATCTT |
| mouse *Pd-l1* | AACGCCACAGCGAATGATGT | ACAGGATGGATCCCAGAAGC |
| mouse *Gzma* | ATGATTTGTGCAGGGGACCT | AGAGGTGATGCCTCGCAAAA |
| mouse *Gzmb* | CCTGCTACTGCTGACCTTGT | GCCATGTAGGGTCGAGAGTG |
| mouse *Prf1* | CCCACTCCAAGGTAGCCAAT | GAGCTGTTAAAGTTGCGGGG |
| mouse *Tnf-α* | CAGCCTCTTCTCATTCCTGC | ATGAGAGGGAGGCCATTTG |
| mouse *Cxcl1* | GGTGTCCCCAAGTAACGGAG | TTGTCAGAAGCCAGCGTTCA |
| mouse *Hif1α* | GGCGAGAACGAGAAGAAAAA | AAGTGGCAACTGATGAGCAA |
| mouse *Vegfα* | GATCATGCGGATCAAACCTC | AATGCTTTCTCCGCTCTGAA |

**Table.S5.Antibodies for Flow Cytometric analysis**

| **Antigen** | **Clone** | **Fluorophore** | **Company** |
| --- | --- | --- | --- |
| CD45 | I3/2.3 | APC.Cy7 | Invitrogen # A15395 |
| CD4 | GK1.5 | PE | eBioscience # 12-0041-82 |
| CD8 | 53-6.7 | FITC | eBioscience # 11-0081-82 |
| NK1.1 | PK136 | PE.Cy7 | eBioscience # 25-5941-82 |
| CD11b | M1/70 | PerCp.Cy5.5 | eBioscience # 45-0112-82 |
| LY6C | HK1.4 | PE.Cy7 | eBioscience # 25-5932-82 |
| LY6G | RB6-8C5 | APC | eBioscience # 17-5931-82 |
| CD206 | MR6F3 | PE | eBioscience # 12-2061-82 |

**Table.S6. Chemokine & cytokine gene names**

Provided as pdf file
