# Supplementary material for: JAK–STAT inhibition impairs K‐RAS‐driven lung adenocarcinoma progression
Source: Int J Cancer. 2019 Sep 10;145(12):3376–88. doi: 10.1002/ijc.32624 (PMC6856680; doi:10.1002/ijc.32624)
Supplement: Supplementary file 3 — Table S6 Chemokine & cytokine gene names [file IJC-145-3376-s003.pdf]

GeneName	ctrl1	ctrl2	ctrl3	ctrl4	Ruxo1	Ruxo2	Ruxo3	Ruxo4	baseMean	log2FoldChange
Ccl12	75	97	66	70	6	14	7	1	41.39710775	-2.544246772
Ch25h	540	698	245	587	135	66	42	16	286.4406661	-1.98601756
Gm12250	347	95	213	369	48	59	21	30	148.8688933	-1.813395035
Gm4951	376	951	141	432	105	79	51	53	267.3841877	-1.779541402
Isg15	401	264	359	398	130	89	66	67	221.3344693	-1.775899959
Ccr1	285	356	767	287	151	65	78	32	248.9477901	-1.710781289
Slc17a6	27	13	55	15	2	3	1	2	14.81540035	-1.638042182
Ifi204	132	105	129	153	49	47	26	24	82.54414019	-1.607410249
Clca1	578	279	861	47	9	56	30	32	238.0195702	-1.567371938
Tfec	159	151	139	186	79	28	30	32	99.85047107	-1.531312984
Alpk2	26	17	17	26	3	3	5	4	12.7004612	-1.528043382
Lama1	305	203	79	183	37	26	28	53	115.7425455	-1.524456175
Rsad2	241	138	1846	375	89	76	57	58	359.5635758	-1.522300146
Ccl7	47	34	45	58	9	7	20	2	27.46431307	-1.485623937
Iigp1	2800	1217	743	2861	691	499	288	301	1177.205432	-1.478744109
Serpina3f	354	81	141	302	46	41	29	53	133.3556126	-1.477525535
AU022793	93	73	52	77	36	16	14	5	45.10411759	-1.476872709
Zbp1	463	275	174	493	134	95	66	84	223.5698934	-1.43583811
Nlrp3	105	80	236	127	46	53	30	15	85.44661286	-1.432741399
Serpina3g	1035	468	354	1221	223	235	118	191	484.3659389	-1.402039843
Pgam2	283	58	69	156	23	50	22	17	85.67872713	-1.398685119
Ffar2	105	109	45	138	33	41	21	14	62.4193314	-1.398046403
Plac8	364	406	542	403	154	178	123	138	287.5203724	-1.39179168
Meg3	7336	45548	5802	5436	3017	1869	1460	2663	8844.214876	-1.385675321
Ncr1	24	12	12	43	4	4	4	2	13.12814454	-1.366539803
Slfn1	116	71	503	140	48	58	37	14	122.1855885	-1.362658625
Gzma	229	73	78	175	28	27	36	40	87.29758043	-1.356925907
Sept3	248	233	268	259	109	127	88	55	171.258165	-1.351105786
Ackr1	22	42	38	23	9	9	8	8	19.68001418	-1.343496826
Slfn2	1031	937	1171	1022	462	556	333	219	707.1524876	-1.336055284
Marco	665	984	1008	932	388	348	345	279	613.0601012	-1.332573437
Cxcl10	503	128	60	475	63	39	27	57	172.1242435	-1.331172407
Cxcl3	83	209	16	103	24	17	14	17	59.23448744	-1.314513678
Ank1	113	171	322	120	63	81	51	28	116.5692953	-1.310989811
Irf7	577	454	532	619	302	181	174	155	372.3859781	-1.30564396
Pim1	780	1104	999	899	404	454	434	202	647.9410312	-1.300589528
Igtp	2345	614	742	1889	575	401	307	301	903.5848049	-1.289212286
Cdkn3	33	70	27	35	14	11	11	11	26.15327141	-1.284524029
P2ry13	160	133	200	241	91	56	38	6	113.3751784	-1.2824205
Mx1	111	230	78	159	69	47	50	20	93.0877141	-1.275008474
Rph3a	69	72	244	47	11	27	37	15	64.82854331	-1.274280597
Lilrb4a	615	605	937	642	378	277	181	206	476.7694587	-1.262139615
Rtp4	493	351	348	510	155	211	196	99	293.0331133	-1.258021306
Ube2c	154	409	264	216	100	107	43	81	169.4529115	-1.250984039
Klrb1c	46	25	16	62	9	12	4	8	22.86508479	-1.24748584
Ifi44	154	163	127	182	79	62	52	51	108.1089048	-1.24391463
Klra7	13	11	9	17	3	1	3	2	7.373129808	-1.236822364
Rab44	260	239	288	250	125	110	111	81	181.8119624	-1.235656476
Mirg	77	768	22	72	29	18	21	11	121.0518121	-1.227431346
Tgtp1	201	139	79	242	74	20	4	23	97.54453014	-1.226252482
Ifit1	548	362	480	612	236	239	202	132	349.0388009	-1.223020587
Chl1	76	555	135	39	37	43	36	24	113.5820614	-1.222272028
Socs3	1415	3485	1556	1386	494	413	503	825	1257.791048	-1.221883283
Ifi47	1299	906	625	1130	459	456	339	291	685.6231402	-1.211347939
Fpr2	354	123	435	379	181	110	75	41	210.4077907	-1.202467257
Cadps	47	26	177	26	12	13	20	5	40.47971013	-1.196066034
Kif5a	474	302	2174	249	128	202	225	119	482.5567234	-1.195355605
Socs1	282	261	110	288	85	76	107	12	149.6479065	-1.19468513
Eef1a2	345	78	533	273	45	137	101	37	193.2988324	-1.185941674
Dctd	26	48	28	14	13	8	6	5	18.12769982	-1.183878966
Irf9	1221	1179	964	1201	682	496	517	246	800.524285	-1.182465614
Ccl3	81	70	19	49	17	16	8	0	31.92589454	-1.178425144
4931408D14Rik	29	63	11	17	9	7	6	4	17.81188524	-1.174819075
Padi4	52	236	337	45	30	35	29	42	99.65357067	-1.168882971
Tgm1	259	468	194	298	160	74	67	110	202.3067277	-1.164660444
Tmem130	58	39	212	35	14	30	22	11	52.2813095	-1.162351445
Slc35f1	53	231	102	41	23	16	19	35	64.18579801	-1.161534125
Gda	887	1055	3885	1198	550	441	399	597	1130.320144	-1.160229409
Cdc25c	23	58	18	20	14	4	7	4	18.01975973	-1.155233174
E230016K23Rik	13	21	10	14	3	3	6	2	8.860607608	-1.152876659
Cdk5r2	25	30	115	21	9	10	15	8	28.94969794	-1.147473153
Apol9a	32	30	20	19	6	6	13	6	16.43483944	-1.14689179
Sst	56	32	140	21	8	13	22	11	37.92410051	-1.143573266
Il18rap	321	1121	515	327	254	222	163	158	376.3445577	-1.133813379
Tnf	97	75	28	77	29	25	20	2	43.29569572	-1.133309831
Eldr	16	16	12	15	7	3	3	3	9.305253129	-1.130956445

Arg1	73	817	72	73	48	46	22	35	141.9386009	-1.130146525
Tnfsf9	1362	1191	315	1184	667	241	198	118	648.9669969	-1.127574047
Irgm1	1757	1301	875	1712	784	692	590	312	990.7894441	-1.124112602
Proz	62	91	17	65	22	22	20	7	37.41312757	-1.122314967
Cntn2	36	11	107	13	7	6	9	1	23.64683917	-1.119513166
Ms4a4c	88	49	102	93	23	48	38	15	56.46699718	-1.119072783
Prg4	12	131	311	28	17	25	15	5	66.38679317	-1.117579365
Serpina3h	62	23	19	30	12	13	10	2	21.24026762	-1.113009593
Cd300lf	337	366	614	279	262	113	63	114	266.4152477	-1.108476249
Apol9b	51	64	34	75	28	20	17	19	38.28262543	-1.107844893
Mobp	23	13	104	8	0	2	5	2	19.65580837	-1.106318783
Pbld2	280	767	159	328	172	122	116	110	251.7232585	-1.106084391
Gbp4	1458	476	595	1282	496	405	323	209	654.4729645	-1.105663333
Syt13	26	29	137	27	7	14	18	9	33.19925196	-1.09913792
Bcl3	563	995	441	750	268	166	250	311	468.3367838	-1.097815966
Usp29	20	11	85	7	4	2	4	4	17.19156334	-1.095955118
Syn1	126	91	363	76	45	45	63	45	106.8130833	-1.092705233
Oasl2	1134	840	1163	1155	578	539	442	382	775.9147595	-1.091877506
Btbd17	28	43	28	19	10	12	7	11	19.66847761	-1.088827999
Rasgrp4	215	128	478	233	79	120	102	82	179.6526276	-1.084945363
Mapk10	91	40	197	85	16	50	42	14	66.40422421	-1.084038525
Saa3	52	319	231	74	56	35	28	54	104.5142526	-1.081076766
Atp1a3	322	285	1522	213	115	157	241	107	368.1719372	-1.078469433
Lilr4b	305	350	579	327	203	159	116	159	274.2919269	-1.073130112
Baalc	45	20	122	12	4	9	15	7	29.3342137	-1.072868126
Tgtp2	209	109	159	251	89	96	75	47	128.557252	-1.071835574
Wfdc21	240	153	626	240	119	89	62	124	208.5403689	-1.07065746
Myc	302	448	278	352	172	177	145	130	248.0411401	-1.069492469
Sema6b	419	593	626	451	260	314	255	136	374.8539412	-1.066778244
Mnda	83	44	26	97	25	28	23	7	41.16585137	-1.066680828
Epb4.2	15	13	133	14	6	7	2	5	24.33738458	-1.065005235
Ccr2	835	661	716	981	415	466	285	58	539.5299004	-1.064458336
Sh2d5	27	38	56	20	12	10	19	7	23.29346459	-1.064104053
Ttk	36	91	32	45	21	24	18	12	34.07019076	-1.063735644
Pcsk1n	88	53	329	31	18	34	39	21	76.49227459	-1.060562547
Ltb4r1	53	98	211	64	45	46	37	22	70.79155574	-1.060378128
Lamp5	7	7	30	5	1	1	2	0	6.559902069	-1.058569075
Ifitm6	12	16	513	40	5	7	10	7	76.08474967	-1.058202167
Scimp	209	151	40	192	70	54	22	39	96.98083389	-1.056746589
Ifi205	111	65	51	139	47	49	19	8	60.13552791	-1.055929006
Tagln3	47	38	104	18	16	15	22	3	32.33655358	-1.055196022
Alox15	23	144	295	11	1	21	4	3	61.41142214	-1.051888985
Gm6116	10	10	8	6	3	0	1	1	4.847874415	-1.050867358
Slc39a2	216	301	105	202	89	51	29	92	136.2245994	-1.050662594
5830416P10Rik	31	69	106	22	18	30	8	5	35.18884661	-1.050204971
Rgs16	130	337	68	154	93	75	39	29	112.1860608	-1.049732068
Lrrc3b	19	25	11	20	8	7	7	3	12.27183064	-1.0466726
3110035E14Rik	28	31	147	20	8	16	14	12	34.43564088	-1.044355635
Wfdc17	246	359	987	179	121	95	61	180	280.907116	-1.042401368
Nrsn1	41	42	192	30	9	20	26	17	47.08308589	-1.040593778
Bcl2l15	19	298	30	13	6	9	6	12	47.0340541	-1.038467449
Nos1	34	21	111	17	6	13	13	10	28.18993596	-1.038135785
S1pr5	36	11	34	34	9	8	14	1	18.19799376	-1.036708918
Gal	144	56	42	123	47	21	20	30	61.00229029	-1.036173244
Ccl2	46	44	69	61	26	15	33	16	38.48353733	-1.034457566
Ankle1	40	73	26	48	19	18	11	19	31.59567252	-1.033218724
Fcgr1	230	210	83	198	122	78	44	38	123.5246126	-1.032689637
Cdh8	11	18	26	8	4	4	3	5	9.864937575	-1.03238637
Cenpk	34	52	21	43	17	16	18	8	25.6333846	-1.025939488
Nos2	226	227	145	229	138	79	67	78	147.8423816	-1.019421511
Tub	71	61	149	38	32	32	36	19	54.25423549	-1.017863165
Fsd1	8	9	22	10	2	1	5	2	7.347840307	-1.01770299
BC030867	34	315	12	37	26	7	5	7	52.78112031	-1.016057782
Fam167b	32	182	5	61	17	10	10	9	39.3797271	-1.015681497
Lrg1	9275	25367	5170	6412	4140	1244	925	4437	7075.824532	-1.0146294
G530011O06Rik	31	41	34	31	24	9	14	8	23.60038567	-1.014293619
Gbp5	740	250	220	774	237	228	184	108	341.9004733	-1.013592403
Kcnj3	87	12	110	47	10	30	19	7	40.27490241	-1.012432706
Svop	33	31	119	18	16	17	15	9	31.95763422	-1.012350904
Slc6a1	65	47	321	28	21	27	38	19	70.4906328	-1.008911043
Cdc20	267	619	107	369	181	151	113	59	226.6949156	-1.007772884
Pilra	319	287	308	294	207	99	71	127	214.0476774	-1.006431901
Iqsec3	23	25	158	14	7	10	18	5	32.21372485	-1.00332812
Dlgap5	40	103	49	77	39	35	24	10	45.68876447	-1.003163371
Csmd3	6	9	44	8	2	3	3	2	9.566179711	-1.002981012
Rpl3l	24	13	9	11	6	2	2	4	8.96421104	-1.000766891
Serpina3i	28	16	19	35	6	5	3	12	15.88292185	-1.000547013

Fam150a	125	162	54	91	37	37	13	50	71.57549043	-0.999940683
Cd209d	14	8	12	6	4	1	1	2	6.028533272	-0.99967683
Sell	212	130	904	228	96	191	123	39	236.9669339	-0.996868234
Ctnna2	17	16	82	13	4	10	9	5	19.39394063	-0.995237635
Dlk1	433	3213	120	169	100	33	53	38	495.5300982	-0.995193586
Fabp7	12	14	33	3	0	1	1	3	8.435770039	-0.995000103
Ptges	974	3720	737	921	671	564	515	485	1047.448671	-0.994103534
Klra2	261	184	303	282	199	103	86	75	184.8514003	-0.993020176
Gbp6	886	286	242	732	235	192	121	211	368.5342932	-0.99262159
Gng3	29	24	71	13	10	13	13	6	22.16320481	-0.992217654
Grik3	25	16	104	6	4	8	5	0	20.78974813	-0.991827726
Gpr35	124	131	111	145	91	74	46	23	91.05483917	-0.9917478
Ncf4	318	233	417	244	135	217	103	92	217.9665104	-0.991670947
Relt	116	117	259	140	58	92	66	55	112.2461469	-0.990734055
Il1f9	24	27	327	36	13	23	22	13	60.31345174	-0.988575758
Fgr	346	280	808	336	254	177	137	157	310.9159919	-0.988040716
Cxcl5	73	262	17	48	21	10	9	29	57.66477675	-0.987948027
Cntnap2	13	30	57	10	4	7	12	4	16.85753572	-0.986629608
AI607873	213	156	215	232	117	124	89	19	142.3001469	-0.986321108
Vat1l	29	20	117	9	4	9	8	10	25.91538287	-0.984652152
Nusap1	93	228	190	150	100	80	32	57	114.495646	-0.984038522
Oas3	150	106	159	174	117	61	23	32	101.4283939	-0.983830963
Amer2	7	18	33	7	4	3	5	0	9.369721887	-0.983049147
Scn1a	16	10	72	11	4	8	6	4	16.32596052	-0.981484505
Aqp9	20	30	106	25	14	13	5	14	28.39434752	-0.980796994
Ndrg4	366	346	1091	220	153	208	252	135	344.0358975	-0.980205298
Ntsr2	15	33	64	11	6	8	12	7	19.30285077	-0.979573451
Ptgds	15	6	36	9	1	4	1	4	9.632599351	-0.979077467
Osmr	1721	2256	1885	2281	877	1093	1096	880	1503.46104	-0.977968405
A330032B11Rik	8	8	2	9	0	1	0	0	3.475414341	-0.977280949
E230029C05Rik	36	31	8	16	4	12	5	4	14.38650471	-0.977127104
B4galt6	253	2514	331	228	177	258	212	156	495.5853105	-0.976765939
Synpr	19	47	79	11	4	11	14	9	24.01521842	-0.976523648
C130050O18Rik	105	96	86	91	76	40	24	26	67.10721907	-0.976492242
Ccnb2	113	353	116	192	90	74	73	74	133.610139	-0.976397588
Rab33a	13	5	13	12	3	4	3	1	6.710410731	-0.970893586
Fcgr3	976	1080	977	926	597	438	421	456	732.0942962	-0.970295297
Gatm	305	348	159	268	151	157	91	96	194.8973861	-0.969396089
Ankrd22	35	353	37	16	28	15	8	15	60.63380594	-0.967425271
Trim30b	32	18	279	43	11	29	18	13	55.18833126	-0.96714306
Klra3	15	13	6	16	6	1	2	3	7.752126839	-0.966939057
Miat	91	75	399	42	27	54	64	18	95.17615402	-0.966542227
Hrh3	12	18	67	10	4	9	8	3	16.16049734	-0.965874214
Hfe2	71	5	129	46	18	17	11	11	38.77107989	-0.964957086
Cd244	148	93	287	158	112	86	53	40	120.6559726	-0.964181823
Cdh3	22	49	63	23	13	14	17	14	26.66122819	-0.962767476
Gpr141	84	69	214	120	67	72	35	13	82.44046749	-0.962605878
Lrrc25	107	125	164	110	90	72	49	28	91.1947029	-0.962098574
Aldh1a3	21	403	47	28	29	18	20	17	69.55087746	-0.962045485
Gad2	49	20	255	19	9	23	23	8	50.52220519	-0.961702926
Emilin2	233	250	1172	275	207	106	107	176	316.9190738	-0.961140047
Tlr6	31	19	61	21	14	14	12	1	21.19496763	-0.961080907
Tpx2	189	416	154	284	166	116	79	90	183.6365922	-0.960223531
Ivl	48	119	32	64	34	16	2	1	38.13898889	-0.958972856
Elfn2	24	23	90	13	14	14	9	5	23.67494979	-0.956241688
Adgrb2	57	65	258	57	28	48	50	20	71.97764079	-0.956240283
Pianp	81	61	137	48	36	46	37	21	57.7720438	-0.954701607
A530064D06Rik	9	10	108	13	6	3	1	5	19.39421699	-0.953267926
Tnnc1	470	15	359	427	51	149	58	29	195.6493582	-0.95286666
Mmp13	109	101	26	41	45	23	12	12	45.43809009	-0.952422809
Inpp5j	81	132	59	78	55	52	25	23	61.78864057	-0.952137106
Xlr3a	26	21	55	62	8	12	27	8	27.19136719	-0.952093296
Svopl	15	169	9	9	10	5	4	2	26.41380643	-0.951417655
Pdxp	89	134	120	109	65	58	67	11	79.20544409	-0.95041668
Mki67	589	1287	590	870	474	479	307	298	601.2989549	-0.949139806
Fam26f	95	63	20	79	24	37	24	6	42.80023529	-0.94891248
Gria4	23	21	105	18	12	14	15	6	26.45152035	-0.948653359
Megf10	37	32	33	18	16	14	7	11	20.94999217	-0.948106189
Snap91	64	47	241	38	15	31	50	15	62.14564785	-0.947635334
Gm5607	8	29	23	7	7	1	4	3	10.0334516	-0.947588755
Unc13c	26	10	135	10	3	5	9	8	25.94968406	-0.943655543
Elavl3	44	35	170	23	12	16	35	4	41.79703467	-0.943623076
Mgl2	676	779	388	527	360	276	225	252	433.0017301	-0.943369592
AF357359	20	234	15	26	26	6	6	4	39.97819421	-0.943006166
Nefh	20	35	36	13	15	11	8	5	17.47700929	-0.942976596
Gpr84	10	15	12	12	6	6	2	0	7.626208527	-0.94147116
Oas2	359	230	199	383	182	165	54	99	208.1184478	-0.941305327

Gm4841	278	71	12	174	24	1	4	23	75.27098906	-0.941018678
Hcar2	313	274	151	287	130	118	128	107	188.2028994	-0.940518937
Gbp10	41	14	5	32	7	7	2	5	14.28379609	-0.940257127
Grin1	62	140	309	35	31	45	64	28	87.96374254	-0.937629626
Knstrn	94	295	66	206	64	71	49	64	112.1930524	-0.937602869
Hrc	208	12	70	136	10	49	22	3	64.1603266	-0.93586012
Glrb	26	29	100	23	7	15	20	12	28.93105349	-0.935618
Selp	37	126	210	80	42	46	44	38	76.99628348	-0.935491081
Lcp2	385	368	457	452	306	262	156	105	305.7399015	-0.933970717
Add2	41	32	269	27	15	21	35	14	56.45611691	-0.933842059
Gbp3	1092	506	486	1119	442	481	319	233	581.7972676	-0.932589824
Nsg2	168	108	475	94	59	94	110	44	142.8257049	-0.93138375
Aldh1b1	98	38	86	75	32	57	17	14	51.62913385	-0.931310399
Arpp21	30	46	148	18	12	17	29	10	38.2857611	-0.928909511
Ube2l6	928	824	3722	1234	648	714	586	623	1160.105439	-0.927516646
Ttc9b	9	6	15	9	2	3	2	3	6.171627523	-0.926169586
Slc6a11	20	16	81	7	5	5	12	4	18.65004661	-0.925460441
Rims1	16	20	78	19	7	7	13	10	21.28203178	-0.925213498
Srl	265	52	292	414	95	133	94	15	167.8886737	-0.924882342
Pbp2	23	153	20	23	17	8	12	14	32.76792103	-0.924217588
Hpca	22	31	147	20	12	16	20	11	34.61571959	-0.923105531
Clec4d	52	75	537	51	47	41	34	45	110.163234	-0.922939584
Rtn4rl2	34	27	94	48	23	22	16	18	35.21060803	-0.922230295
Lmo3	21	24	80	14	13	14	9	1	21.49477411	-0.922179196
Rbfox1	54	53	193	44	21	39	44	18	57.64723795	-0.920537568
Dlgap1	62	67	253	50	27	47	57	17	71.46071885	-0.918826834
Mast1	48	74	100	27	25	31	35	8	42.41485458	-0.918737376
C5ar1	267	180	544	301	220	152	129	90	232.8638504	-0.917464286
Trdn	144	4	53	64	13	23	15	3	40.29037432	-0.917463917
Troap	25	61	16	27	12	15	13	9	21.79632389	-0.917186047
Fcgr2b	655	786	482	599	376	347	264	277	470.1493341	-0.917182144
E2f8	37	92	53	78	48	27	27	12	45.41996195	-0.916314063
Cd200r1	183	154	105	161	118	68	56	48	110.4251653	-0.915172497
Syt1	154	153	681	93	53	116	130	52	177.2425224	-0.913521563
Lmtk3	59	81	237	44	30	46	51	25	70.8151062	-0.912552502
Batf3	122	74	30	92	40	47	29	17	55.81045285	-0.911230681
Sncb	41	27	128	17	11	18	25	9	34.26557306	-0.910792782
Cyp4f18	321	253	543	264	159	157	119	176	250.8294597	-0.910701901
Trim9	39	30	140	24	16	16	24	17	38.27721863	-0.910564496
Adgrb3	18	15	65	10	7	7	11	5	17.14355271	-0.910094607
Trank1	55	42	331	31	18	36	43	18	71.29420664	-0.910040468
Cacna1f	14	10	10	9	5	4	3	2	7.063805385	-0.909617409
Lst1	125	94	110	88	63	28	33	55	75.2687595	-0.90801532
Fam64a	27	111	26	68	27	31	12	3	36.58287107	-0.907019408
Elf3	1468	2884	866	1390	965	702	811	575	1186.895115	-0.906347644
Acsl6	27	26	87	15	11	16	18	5	25.2355875	-0.905753481
5430437J10Rik	59	51	22	44	25	20	17	15	31.44032646	-0.905503946
Cd274	627	322	338	690	331	281	182	148	362.3030049	-0.905487257
Cited4	79	69	84	74	51	51	31	20	56.42965633	-0.905306064
Car9	35	66	29	33	30	19	13	6	27.99151097	-0.904406967
Irgm2	2008	534	764	2003	855	603	536	148	921.0868657	-0.904333784
Nap1l2	24	22	75	19	3	21	11	4	22.07053992	-0.904101742
Tmem91	8	7	25	5	3	3	1	2	6.728796989	-0.904048566
Adra2a	59	112	127	50	23	36	41	41	61.10813839	-0.903778363
Dync1i1	12	18	54	10	4	4	11	5	14.66897731	-0.90369807
Snap25	191	162	841	118	45	119	169	70	213.2529972	-0.901012952
C5ar2	219	284	119	217	154	114	92	38	150.6816236	-0.900987622
Cxcl1	110	162	35	125	47	27	12	50	71.36264256	-0.900845588
Steap4	226	318	915	239	190	195	173	141	297.2005294	-0.900720426
Scn2b	45	44	179	33	23	24	41	14	49.82478655	-0.900486443
Cpne4	15	19	57	9	6	5	8	8	15.91433057	-0.899913847
Kcnn3	72	130	104	111	62	56	52	40	77.31506874	-0.899224522
Csf2	291	312	67	229	178	70	53	18	148.3290472	-0.898462421
Pirb	613	441	1060	562	389	371	227	276	491.3883593	-0.89676555
Bpgm	1114	1180	8907	1467	1027	1170	939	853	2073.724897	-0.896638411
Pacsin1	80	68	236	53	33	56	57	23	74.86841984	-0.896619395
Gjb5	50	138	67	49	27	35	28	35	53.16375394	-0.896042645
Sbno2	1948	2731	2333	2310	1416	1366	1226	966	1767.435128	-0.895355624
Lilra6	8	30	135	22	7	11	9	13	29.35939469	-0.894603816
Gbp11	28	6	14	13	6	5	3	4	9.996816537	-0.893936502
Slc1a2	322	214	1515	163	88	188	269	107	356.4725592	-0.893596154
Rxrg	26	4	42	24	5	8	9	5	15.45669997	-0.893331507
Siglece	93	107	283	87	59	73	23	58	97.9068337	-0.893318155
Il4ra	3013	5363	3344	3216	2502	1729	1682	1589	2771.737422	-0.893171567
Adprhl1	32	6	23	40	6	7	14	2	16.19349604	-0.892416352
Kcnq2	58	51	310	34	21	40	50	18	72.08563996	-0.892323193
Vstm2l	10	16	41	5	5	4	6	3	11.11827336	-0.891602579

Diras1	13	15	62	8	2	5	11	2	14.58581327	-0.891576333
Spi1	925	864	1041	827	652	484	310	398	684.2625002	-0.890282761
Cck	22	23	79	12	5	5	19	6	21.25695843	-0.890061547
Grrp1	522	426	335	499	265	245	306	86	329.105264	-0.889813931
Lgi1	29	23	90	17	11	20	16	6	26.15182883	-0.889738672
Cspg5	73	71	172	43	41	28	56	22	62.55492591	-0.889278152
A730017C20Rik	36	16	45	30	13	19	16	4	22.04786356	-0.887503771
Apitd1	45	63	18	40	19	24	16	12	29.16206318	-0.886497654
Fhl3	92	143	143	111	60	87	61	42	90.93113744	-0.886117189
Adgrb1	57	56	320	34	13	29	62	15	72.57766695	-0.883849176
AI504432	624	447	696	686	409	398	331	137	457.6899977	-0.883757092
Nrxn3	33	36	168	12	7	11	22	16	38.23084416	-0.882385803
Tnni3	1877	118	874	974	175	571	287	199	641.3228172	-0.880370565
Tnfrsf26	69	143	67	51	48	46	34	25	59.05475997	-0.879720646
Fpr1	218	140	403	246	211	110	88	35	177.6382195	-0.879214307
Cd33	130	131	520	179	120	108	71	87	167.6904826	-0.878367172
Tubb3	104	110	175	74	68	57	42	51	84.86547182	-0.878324767
LOC100038947	191	91	161	113	83	47	28	71	99.50990226	-0.875694584
Fam163b	13	6	53	5	1	4	4	3	11.16934727	-0.875536607
Gm12185	124	101	76	147	71	56	47	4	76.17492743	-0.874864045
Ifi27l2a	307	179	307	481	166	131	212	106	235.0715023	-0.874463682
Plau	331	765	172	371	240	209	204	50	282.5325238	-0.874293727
Nfasc	65	44	197	29	25	35	33	24	56.3878115	-0.874025323
Fgf12	72	25	157	76	17	54	35	8	54.76943299	-0.873890352
Mir124a-1hg	18	21	112	15	7	13	15	9	26.13139048	-0.873701957
Sp5	425	565	74	376	252	108	108	21	234.0629368	-0.872962866
Frrs1l	16	22	80	9	5	10	14	2	19.40164529	-0.872719536
Gadd45a	1410	1180	776	1089	902	629	500	240	824.0503065	-0.872680331
Ccl17	383	516	161	306	87	46	18	190	217.698215	-0.872073168
Shcbp1l	28	16	7	22	9	9	2	3	11.90860473	-0.871834735
Kif22	88	205	60	143	80	69	50	26	87.54601713	-0.871432352
Scrt1	14	11	93	7	7	4	8	5	18.58330796	-0.870772896
Cd72	458	457	103	304	171	173	86	125	233.5590441	-0.868504738
Sez6	24	22	176	21	5	19	19	12	37.18322243	-0.868337889
Dscam	14	9	36	15	6	11	4	3	12.1105382	-0.86821674
Bcl11a	55	54	80	64	43	42	17	20	46.25818727	-0.868043473
Arhgap27os3	24	23	28	36	20	16	9	4	19.53906183	-0.867806174
Naip5	234	571	199	223	175	189	96	104	219.0458979	-0.867716677
Rnf112	42	49	201	24	24	25	39	14	51.61773013	-0.867287714
Phgr1	25	152	69	32	19	17	15	32	44.73377779	-0.867163512
Adora2b	916	771	387	943	682	298	275	96	533.2096104	-0.865625517
Gjb3	236	1112	129	278	263	137	106	103	285.5485987	-0.864972751
Prss16	20	39	31	15	7	13	16	7	18.17835749	-0.864435613

GeneName	ctrl1	ctrl2	ctrl3	ctrl4	Ruxo1	Ruxo2	Ruxo3	Ruxo4	baseMean	log2FoldChange	
EDN1	204	205	709	568	1446	715	1450	500	701.1781466	0.825491003	Ruxo_UP
GDF10	220	176	304	257	298	441	468	300	306.0196549	0.562303586	Ruxo_UP
BMP3	346	330	539	418	605	761	914	338	517.5837866	0.543149608	Ruxo_UP
IL17D	110	71	84	122	142	157	169	118	120.9970465	0.510533578	Ruxo_UP
TGFB3	857	687	950	925	1461	1422	1322	824	1037.697659	0.486962917	Ruxo_UP
BMP7	45	54	69	45	75	76	83	72	64.87004754	0.462372952	Ruxo_UP
TGFB2	344	301	493	491	689	545	523	510	485.9508989	0.42174146	Ruxo_UP
BMP5	519	857	704	809	1081	1092	1101	628	830.8647586	0.382215125	Ruxo_UP
CCRL2	367	203	488	416	491	514	607	398	433.3097704	0.359305676	Ruxo_UP
CMTM4	1835	2553	2369	2426	3584	3113	3100	2187	2605.616909	0.354088092	Ruxo_UP
BMP6	373	198	2108	994	1112	2449	2364	394	1194.561665	0.339056069	Ruxo_UP
SECTM1B	54	83	43	33	79	71	37	97	63.41953532	0.338105933	Ruxo_UP
GREM2	516	662	829	615	718	1037	1182	503	741.4365274	0.314517249	Ruxo_UP
CCL24	2	1	5	4	3	7	7	3	3.927925976	0.277660829	Ruxo_UP
CXCL13	74	120	42	129	99	239	124	41	103.8467324	0.25400601	Ruxo_UP
SPP1	1753	2657	3569	1791	3255	4100	3528	1570	2696.777165	0.253594239	Ruxo_UP
THNSL2	281	435	314	226	290	382	321	482	347.6250513	0.246568879	Ruxo_UP
CTF1	169	224	139	169	240	186	182	223	192.4783072	0.241329939	Ruxo_UP
NRG1	39	50	71	36	40	59	62	74	54.89045017	0.237207778	Ruxo_UP
GDF3	6	12	2	10	19	12	4	6	8.585170831	0.194045107	Ruxo_UP
CXCL14	2078	2624	1977	1785	2510	2741	2861	1740	2254.666726	0.189623272	Ruxo_UP
BMP15	2	7	1	3	8	2	0	9	4.190831508	0.171050795	Ruxo_UP
IL10	3	0	0	2	0	6	1	4	2.097570659	0.165640516	Ruxo_UP
WNT2	50	38	183	80	58	160	194	41	97.42409805	0.165005235	Ruxo_UP
IL17B	7	0	3	2	1	6	6	5	3.857791609	0.162761365	Ruxo_UP
CD40LG	6	5	7	8	4	15	10	4	7.18612011	0.154499164	Ruxo_UP
CD70	3	2	1	0	4	6	0	1	2.026585284	0.150405283	Ruxo_UP
INHA	42	63	73	32	59	72	71	40	55.43620384	0.149088039	Ruxo_UP
CXCL12	2901	2331	3321	4297	5439	3984	3411	2177	3408.347431	0.14536131	Ruxo_UP
TNFSF13B	440	303	395	417	457	486	519	301	409.8013573	0.13325475	Ruxo_UP
CCL20	98	63	22	123	58	166	62	71	82.31276206	0.117061888	Ruxo_UP
GREM1	5	23	1	1	4	13	8	11	8.249504179	0.110517701	Ruxo_UP
TNFSF12	617	590	523	591	611	738	756	453	602.307761	0.108481925	Ruxo_UP
TNFSF10	1394	864	1388	2289	2005	2434	2709	217	1586.306712	0.107575022	Ruxo_UP
CXCL2	15	32	35	19	6	11	10	73	27.72765169	0.103895982	Ruxo_UP
ITCH	1241	1632	1764	1676	1976	2020	1896	1090	1628.492619	0.103641968	Ruxo_UP
HMGB1	9163	11560	7792	9268	10114	9948	9892	10057	9740.718392	0.099153424	Ruxo_UP
TXLNA	1705	2121	1988	2129	2343	2381	2401	1403	2022.975113	0.069384147	Ruxo_UP
CCL11	74	53	122	83	83	124	93	60	85.2984084	0.063821855	Ruxo_UP
IL19	0	0	0	1	0	0	0	3	0.627751123	0.062036986	Ruxo_UP
MSTN	2	0	1	0	0	0	6	0	1.086147225	0.052131173	Ruxo_UP
IL17C	0	0	1	0	1	0	0	1	0.402220837	0.050922292	Ruxo_UP
LEFTY1	53	67	75	41	79	74	69	32	59.57851367	0.048805415	Ruxo_UP
AREG	852	1223	567	1035	1132	506	731	1258	931.7929523	0.047532366	Ruxo_UP
JAK1	2901	2903	3860	3507	3883	3938	3638	2413	3333.050564	0.041522595	Ruxo_UP
GDF7	1	0	0	0	1	0	1	0	0.35810073	0.038399647	Ruxo_UP
IL21	0	0	0	7	1	10	0	0	2.095797824	0.031592164	Ruxo_UP
PPBP	112	471	2972	722	322	634	657	2317	1101.028486	0.029008282	Ruxo_UP
WNT7A	304	291	304	301	497	251	298	213	302.4957466	0.028644632	Ruxo_UP
GDF6	15	31	27	52	49	44	22	18	31.32024835	0.027510515	Ruxo_UP
VEGFA	7644	7382	13661	9629	11079	12476	12142	5060	9641.019529	0.022078304	Ruxo_UP
BMP2	180	140	162	190	258	171	170	106	168.9390629	0.016516669	Ruxo_UP
TNFSF4	1	5	0	3	5	3	2	0	2.206789126	0.013862823	Ruxo_UP
CREB3	1778	1841	1534	1837	2112	1745	1821	1440	1749.492124	0.013003686	Ruxo_UP
BMP8A	0	15	6	1	1	10	4	6	5.314394592	0.004002861	Ruxo_UP
IL11	33	25	23	27	31	43	28	10	26.62824138	-0.016423408	Ruxo_DOWN
NODAL	1	0	0	7	2	5	1	0	1.899301522	-0.019079151	Ruxo_DOWN
CCL25	54	47	42	70	49	62	54	46	52.86025014	-0.02112156	Ruxo_DOWN
IL12A	17	4	31	21	12	15	43	5	18.02550979	-0.022868867	Ruxo_DOWN
FGF2	0	29	13	6	11	10	12	11	11.31645378	-0.022993077	Ruxo_DOWN
TNFSF11	20	26	11	33	31	33	21	7	21.8613948	-0.030913275	Ruxo_DOWN
NDP	2	6	7	1	5	4	3	3	3.807427761	-0.038525058	Ruxo_DOWN
IL7	140	187	126	142	171	160	110	134	145.575793	-0.040220917	Ruxo_DOWN
CNIH4	813	1091	822	933	1015	957	924	685	893.8844137	-0.044212675	Ruxo_DOWN
CRLF1	1628	2526	461	1045	1740	1028	745	1623	1361.773285	-0.046365912	Ruxo_DOWN
CXCL16	2983	1905	1594	2682	2582	2172	1918	2134	2255.626469	-0.049204855	Ruxo_DOWN
AIMP1	1108	1112	819	1026	1138	1044	948	821	995.2299728	-0.049241392	Ruxo_DOWN
CMTM3	865	718	599	857	946	867	797	406	740.776836	-0.065605826	Ruxo_DOWN
CX3CL1	775	1863	1056	904	1016	1314	1339	719	1098.244909	-0.069882501	Ruxo_DOWN
YARS	1378	906	970	1160	1360	1143	1053	751	1078.251626	-0.070638156	Ruxo_DOWN
NAMPT	1815	1606	2088	2169	1999	1973	1912	1420	1857.670569	-0.088495992	Ruxo_DOWN
CCL28	2	11	21	22	21	2	2	18	12.70146967	-0.089083737	Ruxo_DOWN
ADIPOQ	86	3	44	57	24	28	112	10	44.75475563	-0.092099064	Ruxo_DOWN
CCL1	37	9	2	20	29	20	5	8	16.01025061	-0.092337758	Ruxo_DOWN
WNT5A	233	727	386	331	398	447	485	231	393.6481853	-0.099892751	Ruxo_DOWN
WNT1	0	1	2	0	0	0	0	0	0.366137388	-0.103477723	Ruxo_DOWN
CNTF	3	0	3	3	2	2	3	0	1.935850651	-0.107441023	Ruxo_DOWN
CSF1	503	404	1241	748	683	817	919	314	687.1178492	-0.116222813	Ruxo_DOWN
BMP8B	0	4	4	1	1	1	2	2	1.879647216	-0.119141733	Ruxo_DOWN
CMTM6	2860	4364	2864	3355	4028	2818	2791	2598	3179.203493	-0.124734888	Ruxo_DOWN
GDF9	8	10	11	19	12	11	16	4	11.0441581	-0.132073466	Ruxo_DOWN

Gpi1	5543	8076	5170	5497	5680	5229	5020	5502	5716.799607	-0.136174394	Ruxo_DOWN
TNFSF13	56	98	75	72	49	72	80	64	70.59270931	-0.141027783	Ruxo_DOWN
CSF3	3	3	1	0	1	0	0	2	1.315264416	-0.141911502	Ruxo_DOWN
GRN	6876	6924	4939	6927	7184	5853	5264	4909	6071.919513	-0.143994967	Ruxo_DOWN
IL17F	1	1	4	0	0	0	2	0	0.979912864	-0.147748955	Ruxo_DOWN
FAM3C	2289	4625	1887	2322	2627	2465	2197	2353	2579.835896	-0.147764684	Ruxo_DOWN
IL16	337	292	523	396	364	541	448	94	361.2117402	-0.150239722	Ruxo_DOWN
CXCL9	1232	312	37	1140	714	696	180	425	594.0018631	-0.159126491	Ruxo_DOWN
CCL5	365	161	86	321	233	302	126	152	217.0594655	-0.167064982	Ruxo_DOWN
IL24	1	2	0	3	0	1	0	1	1.019380445	-0.185507031	Ruxo_DOWN
GDF2	4	7	24	6	8	6	13	4	8.816185277	-0.202507913	Ruxo_DOWN
IL5	1	2	3	0	1	0	1	0	0.965689088	-0.204611569	Ruxo_DOWN
XCL1	39	6	3	23	25	20	3	4	15.08102065	-0.207477906	Ruxo_DOWN
IL12B	110	55	5	94	37	90	41	34	57.82728708	-0.209436555	Ruxo_DOWN
CMTM7	455	392	380	448	377	432	317	313	387.9014798	-0.214884985	Ruxo_DOWN
IL4	2	1	0	11	2	3	0	0	2.31054828	-0.219245094	Ruxo_DOWN
CCL19	157	63	69	103	90	111	75	58	90.19650464	-0.220391196	Ruxo_DOWN
IL13	5	1	0	1	1	1	0	0	1.12167081	-0.224905913	Ruxo_DOWN
CMTM8	1415	2103	672	1208	1176	1016	1013	1124	1215.243772	-0.228629368	Ruxo_DOWN
LIF	224	606	145	218	207	155	166	316	257.8603198	-0.246368547	Ruxo_DOWN
TGFB1	1620	1767	1626	1759	1737	1369	1326	1240	1547.689724	-0.247609268	Ruxo_DOWN
IL6	8	3	2	9	2	0	3	6	4.354758572	-0.251968471	Ruxo_DOWN
MIF	1225	2821	774	1070	1068	896	999	1390	1287.221246	-0.253688224	Ruxo_DOWN
TSLP	61	41	61	58	47	58	65	16	49.53884857	-0.255506765	Ruxo_DOWN
IL2	6	0	3	1	1	2	0	0	1.621632033	-0.256066806	Ruxo_DOWN
CMTM5	6	1	16	1	1	2	4	4	4.494665336	-0.28493591	Ruxo_DOWN
IL17A	2	6	1	0	0	0	1	0	1.205095731	-0.290506908	Ruxo_DOWN
Ccl27a	83	75	50	57	61	55	40	50	58.90520492	-0.306155937	Ruxo_DOWN
TIMP1	295	390	269	273	293	188	134	272	266.3714334	-0.334939159	Ruxo_DOWN
OSM	43	35	125	28	19	19	16	68	46.29378596	-0.342081075	Ruxo_DOWN
BMP1	3154	4810	1727	2812	3348	2332	1772	1936	2702.095206	-0.343214104	Ruxo_DOWN
TNFSF15	107	84	52	130	127	74	60	20	79.17931792	-0.352200164	Ruxo_DOWN
CLCF1	267	514	158	232	218	207	240	180	248.9623688	-0.366548269	Ruxo_DOWN
CCL22	445	410	102	426	255	269	137	266	290.9530704	-0.36794557	Ruxo_DOWN
IL1A	60	87	34	91	78	61	44	17	57.01236149	-0.37282626	Ruxo_DOWN
INHBA	18	49	26	15	12	16	23	19	22.16636134	-0.376352107	Ruxo_DOWN
IL27	12	8	5	13	2	7	4	8	7.568311464	-0.388238706	Ruxo_DOWN
INHBB	908	3985	865	1031	1330	1102	923	1029	1370.19869	-0.39353278	Ruxo_DOWN
SLURP1	55	82	266	114	65	39	162	57	104.1336922	-0.40082447	Ruxo_DOWN
PF4	265	1110	1316	512	219	293	378	813	629.4757859	-0.407183684	Ruxo_DOWN
SP100	842	668	979	909	770	843	712	261	729.5977671	-0.412412628	Ruxo_DOWN
CCL8	185	81	8	219	69	104	36	55	94.78117549	-0.425211749	Ruxo_DOWN
TNFSF8	13	25	16	16	8	21	10	6	14.00825905	-0.439435263	Ruxo_DOWN
IL15	92	108	131	104	86	87	98	46	92.30639677	-0.449760029	Ruxo_DOWN
SCGB3A1	8799	11205	3774	4621	2059	4650	3688	6107	5711.132967	-0.454514978	Ruxo_DOWN
IL1B	153	175	968	109	82	22	66	235	233.4416206	-0.466797648	Ruxo_DOWN
LTB	543	369	285	528	286	476	338	71	351.7563757	-0.480739143	Ruxo_DOWN
IL18	290	207	234	274	204	145	171	159	210.9198092	-0.510132089	Ruxo_DOWN
IFNG	28	8	2	34	13	9	6	3	12.75917566	-0.515026321	Ruxo_DOWN
S100A14	822	2522	729	708	766	421	411	918	914.8927736	-0.519275128	Ruxo_DOWN
GDF11	6	39	21	18	12	21	5	4	15.11806334	-0.523394938	Ruxo_DOWN
TNFRSF11B	61	154	54	88	72	52	65	37	71.22001039	-0.526810832	Ruxo_DOWN
IL34	911	1612	470	795	765	530	423	582	756.0565963	-0.529709459	Ruxo_DOWN
EBI3	122	44	59	76	39	79	38	31	60.75128069	-0.532459343	Ruxo_DOWN
GDF5	17	25	3	14	10	7	8	4	10.76338061	-0.542702119	Ruxo_DOWN
BMP4	2319	6102	1359	2101	2737	1949	1877	460	2263.290134	-0.544325893	Ruxo_DOWN
STAT3	4328	5510	5053	5264	3834	3129	3306	3118	4178.578498	-0.549774626	Ruxo_DOWN
IL1RN	286	336	215	290	257	128	96	187	225.0014107	-0.568621872	Ruxo_DOWN
C1QTNF4	73	124	134	53	56	58	42	65	75.80986726	-0.569193031	Ruxo_DOWN
CXCL11	21	2	3	11	0	2	0	3	5.476121905	-0.583972646	Ruxo_DOWN
TNFSF18	6	70	6	7	10	5	1	0	12.35988113	-0.621541242	Ruxo_DOWN
CCL4	61	30	11	36	22	28	8	3	24.37863618	-0.642550746	Ruxo_DOWN
SCG2	62	57	267	35	12	44	55	48	73.13726607	-0.642664909	Ruxo_DOWN
LTA	39	16	9	48	10	15	12	13	20.47696226	-0.645255582	Ruxo_DOWN
GDF15	433	524	88	395	277	145	89	179	265.5652506	-0.648139232	Ruxo_DOWN
IL23A	10	22	12	17	13	8	6	3	11.029544	-0.657079433	Ruxo_DOWN
THPO	10	148	18	12	14	15	7	14	28.70925282	-0.683367264	Ruxo_DOWN
CKLF	115	159	101	115	88	91	71	46	96.45133946	-0.690528776	Ruxo_DOWN
TNFSF14	23	39	125	26	18	35	24	14	37.42529502	-0.708137304	Ruxo_DOWN
IL33	4712	6413	2227	4736	4462	1990	1758	1564	3418.500286	-0.710086177	Ruxo_DOWN
FAM3B	7	8	24	11	7	4	4	0	7.90546946	-0.778502816	Ruxo_DOWN
CCL17	383	516	161	306	87	46	18	190	217.698215	-0.872073168	Ruxo_DOWN
CSF2	291	312	67	229	178	70	53	18	148.3290472	-0.898462421	Ruxo_DOWN
CXCL1	110	162	35	125	47	27	12	50	71.36264256	-0.900845588	Ruxo_DOWN
CXCL5	73	262	17	48	21	10	9	29	57.66477675	-0.987948027	Ruxo_DOWN
CCL2	46	44	69	61	26	15	33	16	38.48353733	-1.034457566	Ruxo_DOWN
CCR2	835	661	716	981	415	466	285	58	539.5299004	-1.064458336	Ruxo_DOWN
TNFSF9	1362	1191	315	1184	667	241	198	118	648.9669969	-1.127574047	Ruxo_DOWN
TNF	97	75	28	77	29	25	20	2	43.29569572	-1.133309831	Ruxo_DOWN
CCL3	81	70	19	49	17	16	8	0	31.92589454	-1.178425144	Ruxo_DOWN
CXCL3	83	209	16	103	24	17	14	17	59.23448744	-1.314513678	Ruxo_DOWN
CXCL10	503	128	60	475	63	39	27	57	172.1242435	-1.331172407	Ruxo_DOWN

CCL7	47	34	45	58	9	7	20	2	27.46431307	-1.485623937	Ruxo_DOWN
